# Melatonin Mediated Differential Regulation of Drought Tolerance in Sensitive and Tolerant Varieties of Upland Cotton (*Gossypium hirsutum L.)*

**DOI:** 10.3389/fpls.2022.821353

**Published:** 2022-04-04

**Authors:** Laha Supriya, Pullaiahgari Durgeshwar, Mehanathan Muthamilarasan, Gudipalli Padmaja

**Affiliations:** Department of Plant Sciences, School of Life Sciences, University of Hyderabad, Hyderabad, India

**Keywords:** cotton, drought tolerance, melatonin, ROS homeostasis, photosynthesis, nitrogen metabolism, autophagy

## Abstract

Melatonin (*N*-acetyl-5-methoxytryptamine), a biomolecule with multifunctional phyto-protectant activities, enhances the tolerance to broad-spectrum biotic and abiotic stresses in plants. However, little information is available on the effect of melatonin on different morpho-physiological, biochemical, and molecular parameters during drought stress incidence in varieties contrastingly differing in their tolerance levels. The present study is aimed at investigating the drought stress responses of drought-sensitive (var. L-799) and drought-tolerant (var. Suraj) varieties after exogenous melatonin priming and gaining mechanistic insights into drought tolerance in upland cotton (*Gossypium hirsutum*). Melatonin-priming enhanced the tolerance of L-799 to drought stress by modulating the antioxidant system, with increased photosynthetic activity, water-use efficiency, and nitrogen metabolism. Higher endogenous melatonin content and upregulated expression of candidate stress-responsive genes in primed L-799 suggested their involvement in drought tolerance. The higher expression of autophagosome marker [lipidated (ATG8-PE)] in melatonin-primed drought-stressed plants of L-799 also indicated the role of autophagy in alleviating drought stress. Interestingly, melatonin-priming did not show pronounced differences in the different parameters studied during the presence or absence of drought stress in Suraj. In conclusion, this study showed that melatonin plays an important role in mitigating drought stress effects by modulating several physiological, biochemical, and molecular processes, with the key regulatory factor being the plant tolerance level that serves as the switch that turns the priming effects on/off.

## Introduction

The non-availability of water for irrigation causes drought, which is a major limitation to agriculture. The loss inflicted by drought stress in crops is higher than the loss caused by all the pathogenic infections combined (Gupta et al., 2020). Water scarcity affects food crops and economically important species, including cotton (*Gossypium* spp.). *Gossypium hirsutum*, also known as upland cotton, accounts for 90% of global cotton production (Zahid et al., 2021). Decrease in rainfall, groundwater levels, and reduced soil moisture affect the growth, productivity, and fiber quality of cotton (Parida et al., 2007), and it also surges the incidence of diseases, and pest attacks (Zahid et al., 2021). Berry et al. (2013) estimated that the yield loss due to drought in cotton ranges from 50 to 73% globally. Also, drought affects the quality of fiber, causing a heavy toll on the agro-economy in countries like India, which tops the global production of cotton (Parida et al., 2008). Thus, improving the drought tolerance of cotton to withstand water-deficit conditions has become the primary concern of researchers.

The impact of drought on cotton at morpho-physiological, biochemical, and molecular levels was analyzed to understand the complexity of drought tolerance mechanisms (Ullah et al., 2017). Xeromorphic traits and structures conferring tolerance to drought are well-studied in cotton (Iqbal et al., 2013; Ullah et al., 2017). At the biochemical level, phytohormones, including abscisic acid and jasmonic acid, and production of reactive oxygen species (ROS) and ROS redox homeostasis were reported to play roles in conferring drought tolerance (Chen et al., 2013; Ma et al., 2016; Zahid et al., 2021). In addition, genes and regulatory pathways underlying drought stress response were delineated in *G. hirsutum*. Mitogen-activated protein kinases (Zhang et al., 2014; Wang et al., 2016), stress-responsive transcription factors (Hu et al., 2016; Liang et al., 2016), and signaling pathway genes (He et al., 2013; Li L. B. et al., 2015) were extensively studied in *G. hirsutum* for their roles in drought-stress response. The identification of stress-responsive genes facilitated the overexpression of these genes using the transgenic approach for enhancing the drought tolerance in *G. hirsutum* (Parkhi et al., 2009; Yang et al., 2016; Yu et al., 2016). Gao et al. (2017) reported a CRISPR/Cas9 system for editing genes in *G. hirsutum*; however, the application of this approach to improving tolerance to drought stress is yet to be explored. In addition to the aforementioned strategies, biostimulants could be exogenously applied through foliar spray, substrate drench, and seed priming to improve stress tolerance. This approach has been proved to be more sustainable and eco-friendlier to mitigate the adverse effects of environmental stresses. Among different approaches, seed priming is the process of imbibing the seeds with biostimulants to activate specific pre-germination physiological and metabolic states, which impart tolerance to environmental cues (Ashraf and Foolad, 2005; Ibrahim, 2016; Savaedi et al., 2019). Noreen et al. (2013) showed that exogenous application of osmoprotectants (salicylic acid, proline, and glycine betaine) enhances drought tolerance in *G. hirsutum*. Recently, Zhang et al. (2021) have reported the role of melatonin seed priming in enhancing seedling tolerance to salt stress in *G. hirsutum*.

Melatonin (*N*-acetyl-5-methoxytryptamine) is a well-known priming agent that confers multi-factorial protection to the plants against several abiotic and biotic stresses (Zhang et al., 2016; Moustafa-Farag et al., 2020; Turk and Genisel, 2020). Melatonin is considered a better antioxidant than any other classical antioxidants (Hacışevki and Baba, 2018) as it scavenges ROS efficiently. Also, priming with melatonin improved germination, root growth, photosynthesis, and yield (Arnao and Hernández-Ruiz, 2009, 2014, 2019). Melatonin can also stimulate autophagy by regulating the expression of autophagy-activated genes (ATGs), as reported in *Arabidopsis* (Wang et al., 2015) and cassava (Wei et al., 2020). It was found to stimulate plant growth by increasing the uptake and assimilation of nitrogen under high-temperature stress (Zhao N. et al., 2012) and cadmium stress (Erdal and Trunk, 2016). Zhang et al. (2013) reported that melatonin promotes drought tolerance in *Cucumis sativus* and enhances lateral root formation and seed germination. In *Carthamus tinctorius*, melatonin priming improved grain yield, harvest index, and oil yield under drought conditions (Akbari et al., 2020). Seeds of *Brassica rapa* primed with melatonin tolerated drought stress with enhanced morphological traits, seed yield, and seed qualitative attributes (Khan et al., 2019). Melatonin altered the amino acid content in the germinating seeds of *Triticum aestivum* during drought conditions and conferred tolerance to stress (Li et al., 2020). While these reports suggest the positive regulatory effect of melatonin priming to withstand drought stress, no such studies have been carried out to investigate the role of melatonin in imparting drought tolerance in cotton. Moreover, to our best knowledge, there is a lack of information on melatonin effects on drought-distinguished varieties during exposure to drought stress in relation to plant growth, photosynthesis, nitrogen metabolism, and autophagy. Thus, the present study was focused on understanding how melatonin priming influences drought tolerance in drought-sensitive and -tolerant varieties under drought stress conditions and the underlying effects on morpho-physiological, biochemical, and molecular processes in cotton.

## Materials and Methods

### Experimental Materials and Seed Priming

Seeds of two varieties, *viz.*, L-799 (drought sensitive) and Suraj (drought tolerant), were obtained from Regional Agricultural Research Station, Guntur, Acharya N. G. Ranga Agricultural University (ANGRAU), Andhra Pradesh, India, and ICAR-Central Institute for Cotton Research, Maharashtra, India, respectively. The seeds of two varieties were surface sterilized and imbibed with different concentrations (5, 10, 25, 50, and 100 μM) of melatonin (Sigma-Aldrich, United States) for 24 h in the dark. The seeds imbibed in deionized water served as control. The seeds were then dried by placing in an incubator at 25 ± 2°C for 24 h before germination. The control and treated seeds were then placed on sterile germination paper in Petri dishes and allowed to germinate in the dark at room temperature. The germinated seeds were then transplanted onto pots (7 cm) containing a mixture of autoclaved soil, manure, and sand (4:1:1 ratio). The plants were allowed to grow in a culture room at 25 ± 1°C, 65 ± 2% relative humidity under 16:8-h light and dark photoperiod. Two-week-old plants (with two fully grown leaves and an emerging third leaf) were imposed on drought stress by withholding the irrigation until the soil moisture content reduced to 20%, which took another 8 days. The fully grown leaf samples were further used for various experiments. All the experiments have been carried out with three independent batches of plants, with triplicates per treatment in each batch of plants.

### Measurement of Relative Water Content and Reactive Oxygen Species

The fully expanded youngest leaves (true third leaf) after stress treatment were harvested, and relative water content (RWC) was determined by following the protocol of Huang et al. (2019). Histochemical detection of superoxide anion radical (O_2_^–^) and hydrogen peroxide (H_2_O_2_) was performed using nitrogen blue tetrazolium (1 mg ml^–1^) and 3,3′-diaminobenzidine stains (0.5 mg ml^–1^), respectively (Ramel et al., 2009). The H_2_O_2_ and O^2–^ contents were determined according to the method described by Okuda et al. (1991) and Elstner and Heupel (1976), respectively.

### Determination of Melatonin Content

A direct sample extraction procedure was followed to determine the melatonin content. Fresh leaf samples (a true third leaf) were weighed and sliced into small (3–5 mm) pieces, dipped into vials containing chloroform, followed by overnight shaking at 4°C. The leaf disks were then discarded, and the solvent in the vial was evaporated under N_2_ gas at 4°C. The residue was then dissolved in acetonitrile, filtered through a 0.2-μm PVDF membrane filter, and used for HPLC analysis. Shimadzu HPLC system (Kyoto, Japan) with a C18 column (Phenomenex KINETEX 250 mm × 4.6 mm) was used for determining the melatonin content. The mobile phase used was water and acetonitrile (50:50) with a 1-ml- min^–1^ flow rate. The detection was carried out at 280 nm, UV. The melatonin level was quantified in different samples using pure melatonin (Sigma-Aldrich, United States) as a standard by following the method of Arnao and Hernández-Ruiz (2009) with some modifications.

### Assay of Antioxidant Enzyme Activities

Frozen leaf samples (a true third leaf) (150–200 mg) were used to extract the crude protein for enzyme activity assays, following the procedure of Cherono et al. (2020). Superoxide dismutase (SOD, EC 1.15.1.1) activity was determined using nitro blue tetrazolium (NBT), following Beauchamp and Fridovich (1971). Catalase (CAT, EC 1.11.1.6) activity was measured spectrophotometrically according to the method described by Patterson et al. (1984). Ascorbate peroxidase (APx, EC 1.11.1.11) and guaiacolperoxidase (GPoX, EC 1.11.1.7) activities were determined using the methods described by Nakano and Asada (1981) and Polle et al. (1994). Similarly, the activities of monodehydroascorbate reductase (MDAR, EC 1.6.5.4) and dehydroascorbate reductase (DHAR, EC 1.8. 5.1) were determined by the methods of Hossain et al. (1984) and Dalton et al. (1986). Glutathione reductase (GR, EC 1.8.1.7) activity was measured as described by Foyer and Halliwell (1976) with minor modifications.

### Determination of Non-enzymatic Antioxidants, Electrolyte Leakage, Lipid Peroxidation, and α-Tocopherol Levels

The leaf samples (true third leaf) (0.1 g) were homogenized in 5% metaphosphoric acid, followed by centrifugation (20,000 *g* for 15 min at 4°C). Reduced ascorbate and glutathione contents were estimated following the standard procedures of Zhang and Kirkham (1996). Electrolyte leakage was estimated by the method of Dionisio-Sese and Tobita (1998). Electrical conductance (EC) was measured using a conductivity meter (Hanna Instruments, India). A lipid peroxidation assay was performed following the procedure of Heath and Packer (1968). α-tocopherol was extracted from the samples as per the procedure described by Munné-Bosch et al. (1999). The analysis was performed in the HPLC (Shimadzu, Japan) with the following conditions: mobile phase methanol (100%), flow rate (1 ml/min^–1^), and injection (20 μL). The separation was done on a C18 column (Phenomenex, United States), and α-tocopherol was measured at a wavelength (λ) of 295 nm by the DAD. Pure (±) α-tocopherol was used as a standard.

### Measurement of Photosynthetic Parameters, Chlorophyll Content, and Soluble Sugar Content

Leaf gas exchange parameters [net photosynthetic rate (P_n_), stomatal conductance (g_s_), intercellular CO_2_ (C_i_), and transpiration rate (E)] were measured in the fully expanded third leaf after the 8th day of drought stress using a portal infrared CO_2_/H_2_O gas analyser (IRGA; ADC Bioscientific, United Kingdom). The saturating photosynthetically active radiation (PAR) of 1,600 μmol m^–2^s^–1^, temperature (24 ± 2°C), relative humidity (55–60%), and CO_2_ concentration (360 ± 10 μmol mol^–1^) were constantly maintained throughout the measurements. Water use efficiency (WUE_i_) was calculated as a ratio of net photosynthetic rate to the transpiration rate (P_n_/E). The concentration of chlorophyll *a* was measured in a fully expanded true third leaf after the 8th day of drought stress using Dual-PAM 100 (Waltz, Germany). Other photosynthetic parameters measured were Photosystem II yield (Y II), electron transport rate (ETR II), photochemical quenching (qP), non-photochemical quenching (NPQ) regulated heat dissipation [Y(NPQ)], and non-regulated heat dissipation [Y(NO)]. The plants were dark adapted for 20 min before the analysis. The chlorophyll content was estimated using the protocol of Lichtenthaler and Wellburn (1983). The soluble sugar content was estimated by Anthrone method (Li, 2000).

### Assay of Enzymes Related to Nitrogen Metabolism and Measurement of Nitrate, Nitrite, and Ammonium Contents

The activity of four enzymes involved in nitrogen metabolism, namely nitrate reductase (NR, EC 1.6.1.1), nitrite reductase (NiR, EC 1.7.7.1), glutamine synthetase (GS, EC 6.3.1.2), and glutamate synthase (GOGAT, EC 1.4.7.1) was analyzed following Erdal (2019). Nitrate content was determined by reducing it to nitrite and measured spectrophotometrically using a standard curve constructed with known concentrations of KNO_3_ (Cataldo et al., 1975). The nitrite content was determined spectrophotometrically by following the method of Barro et al. (1991). Similarly, ammonium content in the samples was measured using a standard curve constructed with known concentrations of (NH_4_)_2_SO_4_ by following the method of Barro et al. (1991).

### RNA Isolation, cDNA Synthesis, and Quantitative Real-Time PCR

Total RNA was extracted from the leaves of different samples using the CTAB-ammonium acetate method (Zhao L. et al., 2012). RNA integrity was analyzed by gel electrophoresis and NanoDrop 2000 UV-Vis spectrophotometer (Thermo Scientific, United States). Primers targeted for the genes-encoding enzymes involved in ROS metabolism [respiratory burst oxidase protein D (RBOH D), Cu/Zn superoxide dismutase (Cu/Zn-SOD), Mn superoxide dismutase (Mn-SOD), catalase (CAT), cytosolic ascorbate peroxidise (cAPX), Peroxisomal/glyoxisomal ascorbate peroxidase (gAPX), and glutathione reductase (GR)], photosynthetic electron transport chain [photosystem II D1 (PSII D1), plastocyanin (PC), ferredoxin (PET F), *large RUBISCO subunit* (rbcL), fructose-1,6-bisphosphatase (FBP) and sedoheptulose-1,7-bisphosphatase (SBP)], nitrogen metabolism [nitrate reductase (NR), nitrite reductase (NiR), glutamine synthase (GS), and glutamate synthase (GOGAT)] and autophagy [target of rapamycin (TOR), autophagy-related proteins (ATG), likewise ATG2, ATG9, ATG18a, ATG5, ATG12, ATG7, ATG8c, ATG8i, and constitutively stressed 1 (COST1)] were synthesized using GenScript^1^. cDNA was synthesized from the total RNA using Primescript 1st Strand Synthesis Kit (Takara Bio Inc., Japan). Real-time PCR was performed on Mastercycler Realplex (Eppendorf, Germany) in a final reaction volume of 10 μL containing 1 μL of the diluted cDNA, 1 μL of 10 pmol primers (Supplementary Table 1), and 5 μL of SYBR Premix Ex Taq II (TliRNase H Plus) with 0.2 μL ROX (6-carboxy-X-rhodamine) (Takara Bio Inc., Japan). The PCR conditions consisted of initial denaturation at 95°C for 3 min, 40 cycles of amplification [95°C for 30 s, annealing temperature for 20 s, and 72°C for 30 s], and a final elongation stage at 72°C for 5 min. *Actin* was used as an internal control, and the relative fold-change RNA expression was estimated using the ΔΔCT method (Livak and Schmittgen, 2001).

### Total Protein Extraction and Immunoblot Analysis

A fresh leaf sample was macerated in liquid N_2_ and homogenized in a buffer containing 50-mM Tris–HCl pH 8.0, 150-mM NaCl, 1-mM phenyl-methanesulfonyl fluoride, and 10-mM iodoacetamide) (Chung et al., 2009) and protease inhibitor cocktail. For immunoblot analysis, 15% of SDS-PAGE gel was prepared with 6-M urea (Wang et al., 2019). Following electrophoresis of the samples, the protein-containing SDS-PAGE gel was transferred to a nitrocellulose membrane. The levels of ATG8 and ATG8-PE were determined using an anti-ATG8 antibody (AS14 2769, Agrisera, Sweden) in 1:500 dilution. Histone H3 was used as a loading control. Thus, for determining the His-H3 level, anti-histone-H3 (AS10710, Agrisera, Sweden) was used as primary antibody in 1:2000 dilution. An anti-rabbit antibody conjugated to HRP was used as the secondary antibody.

### Statistical Analysis

The data obtained are a mean value of 3 independent treatments with three replicates per treatment in each experiment, which were subjected to one-way ANOVA. The error bars shown in the graph are standard deviation (± SD) of mean values. The significance of differences between treatments was evaluated using Duncan multiple range test (*p* < 0.05).

## Results

### Effect of Melatonin on Drought Tolerance in Sensitive and Tolerant Varieties

Priming the seeds of drought-sensitive and -tolerant varieties with different concentrations of melatonin showed that 10 μM was more effective in mitigating the drought stress effects in L-799 as reflected from highest plant height, root length, and leaf area than other concentrations (25, 50, and 100 μM) as compared to unprimed plants under stress conditions. Interestingly, in Suraj, melatonin at the concentrations tested did not have any significant effect on plant height and root length in drought-stressed plants except the leaf area, which showed a significant increase at 10, 25, 50, and 100 μM compared to unprimed stressed plants (Supplementary Figure 1). Thus, 10-μM melatonin was used to prime the seeds of drought-tolerant and -sensitive varieties in further studies for investigating the role of melatonin in drought tolerance in cotton.

**FIGURE 1:**
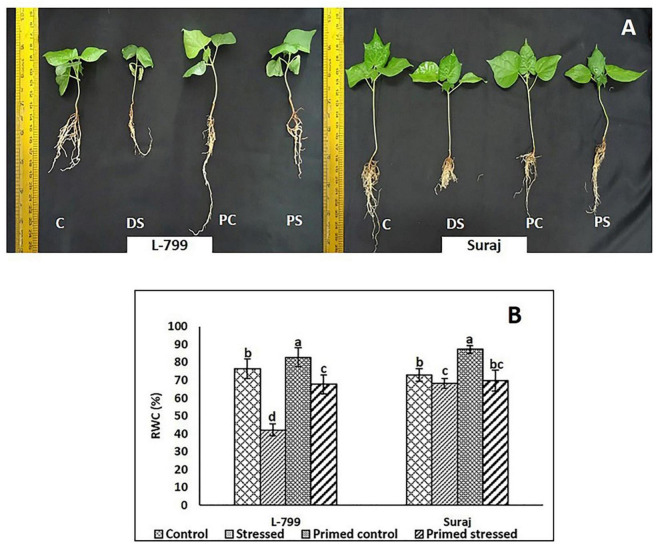
Phenotypic changes in melatonin primed and unprimed plants of drought-sensitive (L-799) and -tolerant (Suraj) varieties of cotton (*Gossypium hirsutum*) under optimal or drought-stress conditions. **(A)** C, Plants grown under optimal conditions (control). DS, Plants grown by withholding water for 8 days (drought-stressed). PC, Melatonin primed plants grown under optimal conditions (primed control). PS, Melatonin primed plants grown by withholding water for 8 days (primed stress), and **(B)** Relative water content (%) in leaves of L-799 and Suraj under drought stress with and without melatonin priming. Data represent mean values ± SD of 3 independent experiments, with 3 replicates per treatment in each experiment. Different alphabets within the group represent significant differences among the treatments according to Duncan multiple range test at *P*<0.05.

Melatonin-primed and control (unprimed) plants of sensitive and tolerant varieties were subjected to drought stress for 8 days, and the changes in morphological parameters were examined (Figure 1A). The control plants of sensitive variety (L-799) showed severe wilting symptoms, whereas melatonin-primed plants were healthy when exposed to drought stress. In drought-tolerant variety (Suraj), the phenotypes of melatonin-primed and unprimed (control) plants were similar in both drought-stressed and control conditions. This distinctly showed that melatonin considerably improved the drought tolerance ability of L-799, whereas it does not have any notable effect on the tolerant variety, Suraj. To further validate the phenotypic observations, relative water content (RWC) was measured in all the samples. The results showed that RWC in L-799 decreased significantly under drought conditions, but the content in melatonin-primed stressed plants was significantly higher than in the unprimed stressed plants (Figure 1B). Although Suraj showed a significant decrease in RWC under stress compared to the control, the content was higher than L-799-stressed plants. Unlike L-799, priming did not significantly change RWC in Suraj compared to unprimed plants under drought stress.

### Effects of Melatonin on Reactive Oxygen Species Accumulation and Detoxification

**FIGURE 2:**
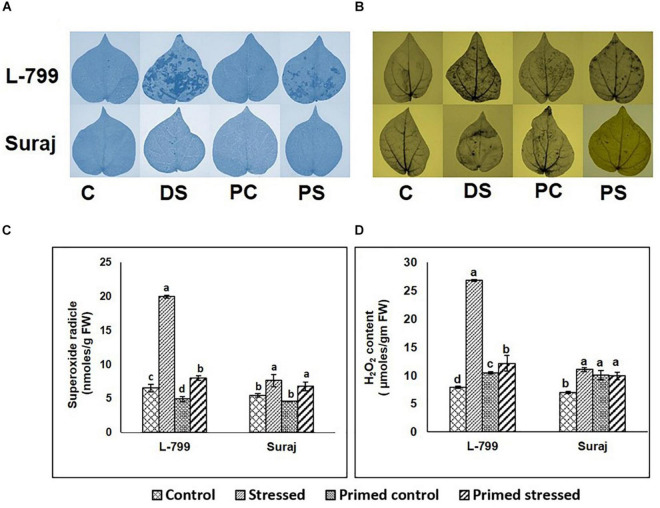
Effect of melatonin on the accumulation of superoxide radicals and hydrogen peroxide in the leaves of drought-sensitive and -tolerant varieties after different treatments. **(A,B)** Superoxide radical and hydrogen peroxide accumulation in the leaves of different treatments visualized by NBT and DAB staining, respectively, and **(C,D)** Quantification of superoxide radicals and hydrogen peroxide levels in leaves of drought-sensitive and -tolerant varieties. C, Plants grown under optimal conditions (control). DS, Plants grown by withholding water for 8 days (drought stressed). PC, Melatonin primed plants grown under optimal conditions (primed control). PS, Melatonin primed plants grown by withholding water for 8 days (primed stress). Data represent mean values ± SD of 3 independent experiments, with 3 replicates per treatment in each experiment. Different alphabets within the group represent significant differences among the treatments according to Duncan multiple range test at *P*<0.05.

NBT and DAB staining showed a higher accumulation of O_2_^–^ and H_2_O_2_, respectively, in the unprimed L-799 than Suraj during drought stress (Figures 2A,B). A relatively lesser accumulation of O_2_^–^ and H_2_O_2_ was observed during stress conditions in melatonin-primed L-799. Quantification showed at least a threefold and fourfold increase in O_2_^–^ and H_2_O_2_ content in the unprimed L-799 during drought stress, respectively (Figures 2C,D). The melatonin-primed L-799 showed significantly reduced levels of O_2_^–^ and H_2_O_2_ when exposed to drought stress, unlike the tolerant Suraj variety, where the levels of unprimed and primed stressed plants remained similar.

**FIGURE 3:**
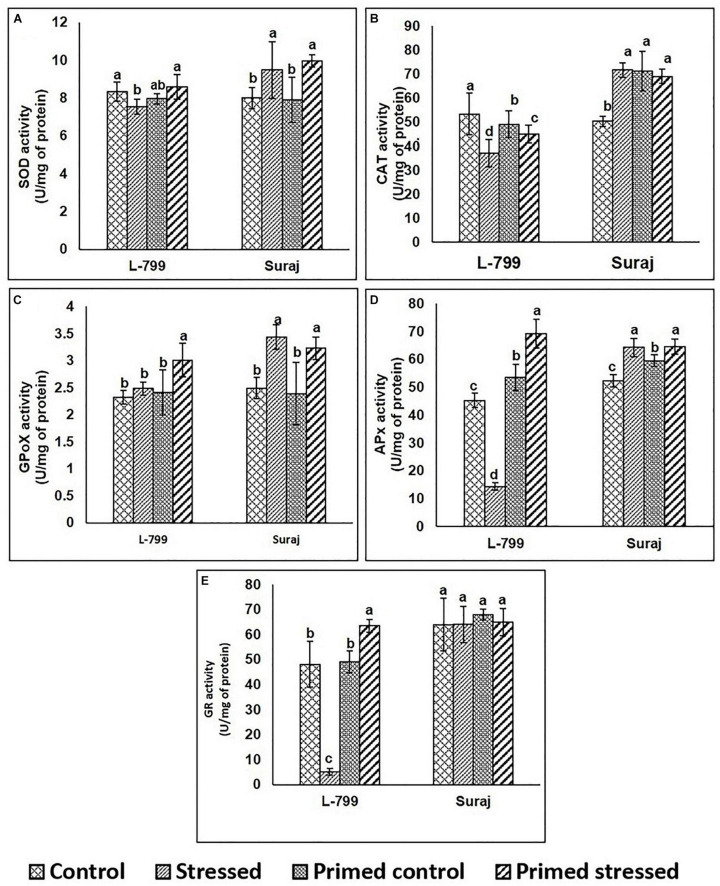
Effect of melatonin priming on antioxidant enzyme activities in drought-tolerant and drought-sensitive varieties with and without drought stress. **(A)** SOD activity, **(B)** CAT activity, **(C)** GPoX activity, **(D)** APx activity, and **(E)** GR activity. Data represent mean values ± SD of 3 independent experiments, with 3 replicates per treatment in each experiment. Different alphabets within the group represent significant differences among treatments according to Duncan multiple range test at *P*<0.05.

To gain further insights into the ROS detoxification mechanism, the activity of enzymes involved in this process was assayed (Figures 3A–D). In the case of SOD, there were no significant differences in the activity of primed L-799 controls and stressed, whereas it was higher in primed Suraj under drought stress than primed controls (Figure 3A). The levels of CAT were comparatively lower in all L-799 samples than Suraj, except the controls of unprimed plants (Figure 3B). GPoX activity was increased in primed stressed plants of L-799, and primed and unprimed plants of Suraj during drought stress (Figure 3C). In the cases of APx and GR, the enzyme activities were significantly reduced in L-799 during drought stress. But primed stressed plants showed the highest activity among all L-799 samples. Unlike L-799, Apx and GR activity was similar in unprimed and primed stressed plants of Suraj (Figures 3D,E).

**FIGURE 4:**
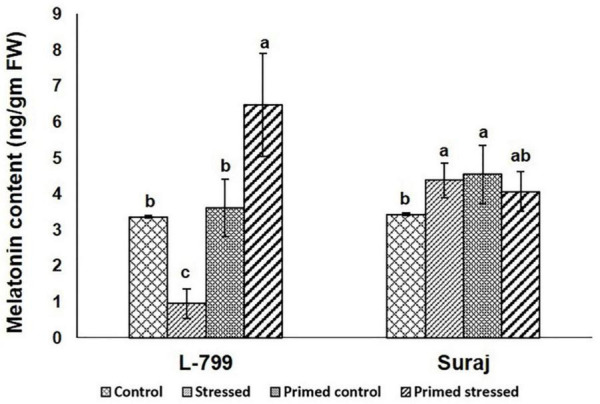
Effect of melatonin priming on endogenous melatonin content in drought-tolerant and -sensitive varieties with or without drought stress. Data represent mean values ± SD of 3 independent experiments. Different alphabets within the group represent significant differences among treatments according to Duncan multiple range test at *P*<0.05.

Furthermore, the endogenous melatonin content was estimated in all the samples (Figure 4 and Supplementary Figure 2). The results showed a drastic increase in melatonin levels during drought-stressed conditions in primed L-799 compared to unprimed plants during drought. Unlike L-799, drought caused an increase in melatonin levels in the unprimed Suraj, with the levels being similar to primed plants under drought stress and unprimed stressed plants.

### Effect of Melatonin on Ascorbate-Glutathione Recycling Reactions

**FIGURE 5:**
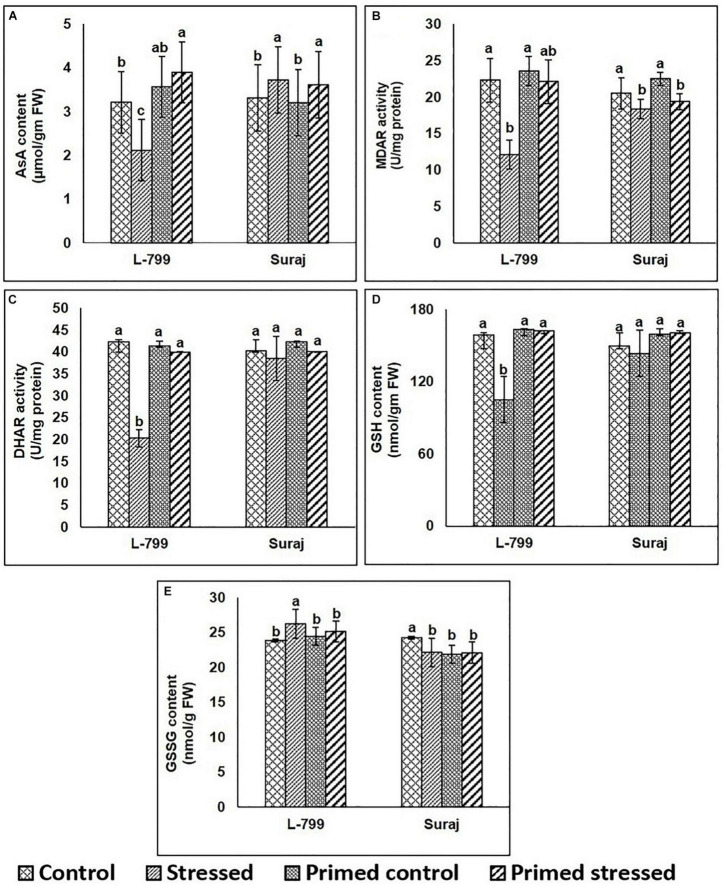
Effects of melatonin priming on antioxidant levels in drought-tolerant and drought-sensitive varieties with or without drought stress. **(A)** AsA content, **(B)** MDAR activity, **(C)** DHAR activity, **(D)** GSH, and **(E)** GSSG contents in leaves of melatonin primed and unprimed L-799 and Suraj varieties after exposure to drought stress or optimal conditions. Data represent mean values ± SD of minimum 3 independent experiments. Different alphabets within the group represent significant differences among treatments according to Duncan multiple range test at *P*<0.05.

The contents of AsA and GSH as well as the activity of MDAR and DHAR increased in both primed (control and stressed) compared to unprimed stressed plants of L-799 (Figures 5A–D). Drought stress caused an increase of the GSSG level in unprimed L-799 compared to other conditions, whereas no change was observed in primed and unprimed Suraj plants under stress conditions (Figure 5E). In Suraj, the AsA and GSH contents, and MDAR and DHAR activities remained similar in primed plants compared to their respective unprimed plants subjected to drought.

### Effect on Electrolyte Leakage, Lipid Peroxidation, and α-Tocopherol Contents

**FIGURE 6:**
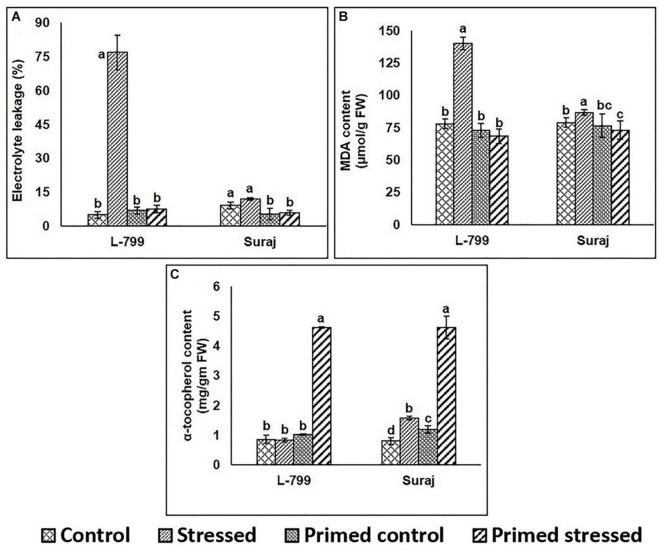
Effect of melatonin priming on electrolyte leakage, lipid peroxidation, and α-tocopherol contents in drought-tolerant and -sensitive varieties with or without drought stress. **(A)** Electrolyte leakage, **(B)** lipid peroxidation, and **(C)** α-tocopherol contents in leaves of different treatments. Data represent mean values ± SD of minimum 3 independent experiments. Different alphabets within the group represent significant differences among treatments according to Duncan multiple range test at *P*<0.05.

A marked increase (14-fold) in electrolyte leakage during drought stress was observed in unprimed L-799, whereas the controls (unprimed and primed) and primed stressed plants did not show any notable differences. Suraj primed plants (both control and stressed) showed a significant decrease compared to unprimed controls (Figure 6A). Drought stress significantly increased MDA content in unprimed stressed plants of L-799, whereas it decreased in primed stressed plants and was comparable to primed unstressed (control) plants. Although Suraj also exhibited higher MDA content under drought in unprimed plants, the levels were lesser than L-799. Primed stressed plants showed a significant change in MDA content compared to unprimed stressed plants (Figure 6B). The α-tocopherol level showed a significant increase in both the varieties under drought stress in primed plants compared to other treatments (Figure 6C and Supplementary Figure 3).

### Effect of Seed Priming on Photosynthetic Leaf Gas Exchange Parameters, Photosynthetic Efficiency, Chlorophyll a Fluorescence Parameters, Total Chlorophyll, and Soluble Sugar Contents

**FIGURE 7:**
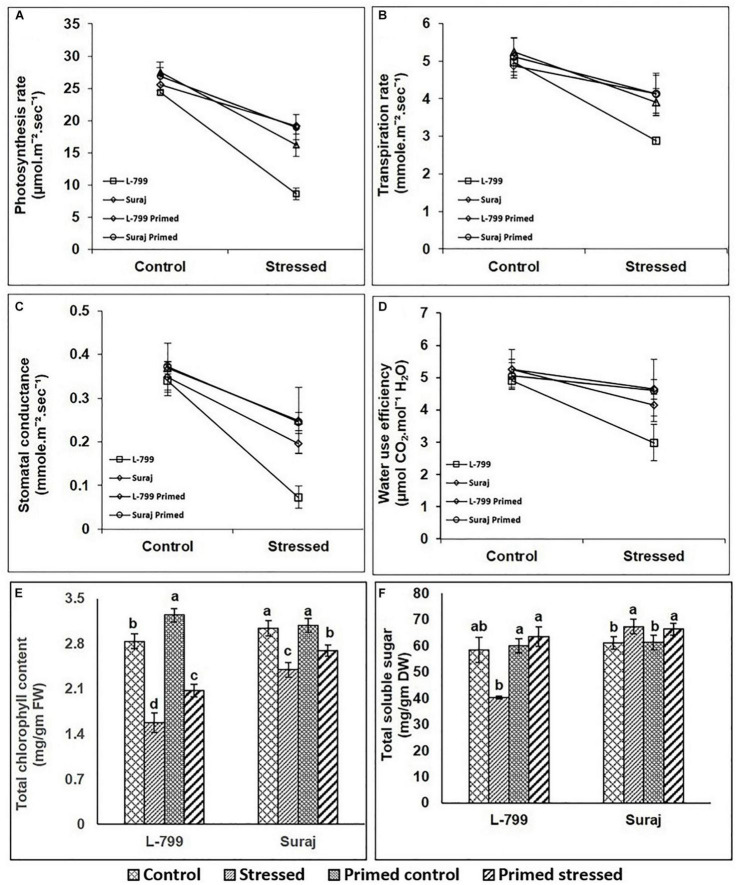
Effect of drought stress on photosynthetic leaf gas exchanges parameters **(A–D)** in drought-sensitive and -tolerant varieties with or without melatonin priming. **(A)** Photosynthetic rate, **(B)** transpiration rate, **(C)** stomatal conductance, **(D)** water use efficiency, **(E)** chlorophyll content, and **(F)** total soluble sugar. Data represent mean values ± SD of minimum 3 independent experiments, with 3 replicates per treatment in each experiment. Different alphabets within the group represent significant differences among treatments according to Duncan multiple range test at *P*<0.05.

The photosynthesis rate (Pn) was significantly decreased by 64.58% in unprimed L-799 under drought, whereas priming showed a significant increase by 121.8% compared to unprimed plants under stress. Suraj also displayed a decrease of 41.19 and 29.3% in stressed and primed stressed plants compared to controls (Figure 7A). Drought stress decreased the transpiration rate of unprimed L-799 by 41.73% compared to the controls, while priming resulted in a 42.56% increase compared to unprimed stressed plants. Suraj showed a 25.52 and 21.52% decrease in stressed and primed stressed plants compared to unprimed controls (Figure 7B). Stomatal conductance of L-799 decreased drastically by 78.5% during drought stress in unprimed compared to controls, but primed stressed showed a 167.4% increase compared to unprimed stressed plants. In Suraj, the stomatal conductance decreased by 32.27% in stressed plants during drought, and melatonin priming was found to have a non-significant effect under stress (Figure 7C). WUE reduced significantly in unprimed L-799 under drought stress, while the changes were non-significant in primed control and primed stressed plants compared to controls. Suraj maintained similar levels of WUE in all samples (Figure 7D). Unprimed and primed stressed plants of L-799 showed a decrease (21 and 11.2%, respectively) in total chlorophyll content compared to the unprimed controls. Although a similar trend was observed in Suraj, the values were comparatively higher than L-799 (Figure 7E). The *PAO* (Pheophorbide a oxygenase a chlorophyll degrader) transcripts level was downregulated in L-799 primed stressed plants compared to unprimed stressed plants (Supplementary Figure 4). Similarly, in Suraj, the transcript levels of PAO were downregulated in primed stressed plants than unprimed stressed plants, although the transcript levels of unprimed stressed plants remained very low as compared to unprimed stressed plants of L-799. Total soluble sugar content decreased significantly in unprimed stressed L-799 plants but increased with priming during drought (Figure 7F). In Suraj, there was no significant change in total soluble sugar content between unprimed and primed stressed plants.

**FIGURE 8:**
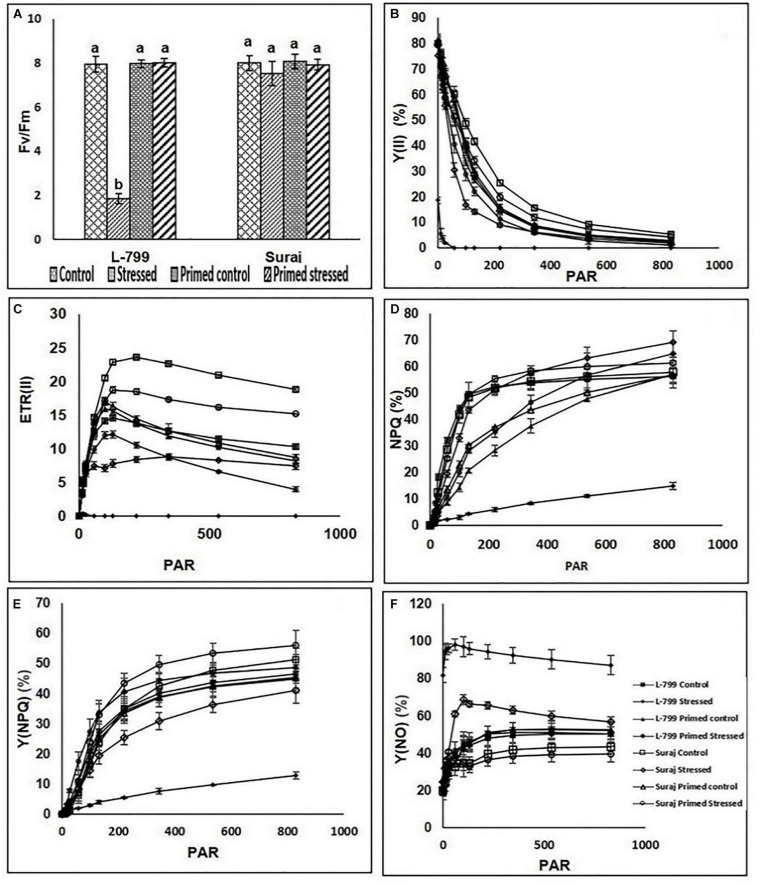
Changes in maximum photosynthetic efficiency of PSII (Fv/Fm) and rapid light curves of photosynthetic parameters **(A–F)** in melatonin primed or unprimed drought-sensitive (L-799) and -tolerant (Suraj) varieties of cotton in optimal conditions (control) or exposure to drought stress. **(A)** Fv/Fm, **(B)** effective photochemical quantam yield [Y (II)], **(C)** electron transport rate of PSII [ETR (II)], **(D)** Non-photochemical quenching, **(E)** non-regulated heat dissipation [Y (NO)], and **(F)** regulated heat dissipation. PAR is photosynthetically active radiation. Data represent mean values ± SD of 3 independent experiments, with 3 replicates per treatment in each experiment. Different alphabets within the group in panel **(A)** represent significant differences among treatments according to Duncan multiple range test at *P*<0.05.

Drought stress dramatically decreased the maximum quantum yield (F_v_/F_m_) in L-799, followed by a pronounced decrease in photosystem II yield [Y(II)], which declined drastically from ≥ 58 PAR (Figures 8A,B). Suraj maintained constant F_v_/F_m_ levels in all conditions. Priming elevated Y(II) values significantly in stressed plants compared to unprimed stressed of both the varieties, more prominently in L-799 (Figure 8B). The electron transport rate (ETR) decremented from ≥ 11 PAR under stress conditions in L-799, whereas unprimed stressed plants of Suraj also showed a decrease in ETR values, although from ≥ 100 PAR. Primed stressed plants exhibited higher ETR (II) than unprimed stressed in both varieties but more significantly in L-799 (Figure 8C). Non-photochemical quenching (NPQ) and regulated heat dissipation [Y(NPQ)] levels were decreased dramatically in stressed plants of L-799 compared to unprimed control plants, but priming significantly elevated both the parameters under stress compared to unprimed stressed plants. Unlikely, in Suraj, unprimed control, unprimed stressed, and primed stressed plants exhibited an almost equivalent level of NPQ (Figures 8D,E). Non-regulated heat dissipation [Y(NO)] levels increased more significantly in unprimed stressed plants of L-799 than controls (Figure 8F). Priming significantly decreased the Y(NO) level under stress compared to unprimed stressed plants. Although Suraj showed a significant increase in the Y(NO) level in unprimed stressed plants from ≥ 58 PAR compared to control plants, the values were considerably lesser compared to L-799 stressed plants, and priming aided a significant decline from ≥ 58 PAR onward (Figure 8F).

### Effect of Melatonin on the Nitrogen Cycle and Related Enzymes

**FIGURE 9:**
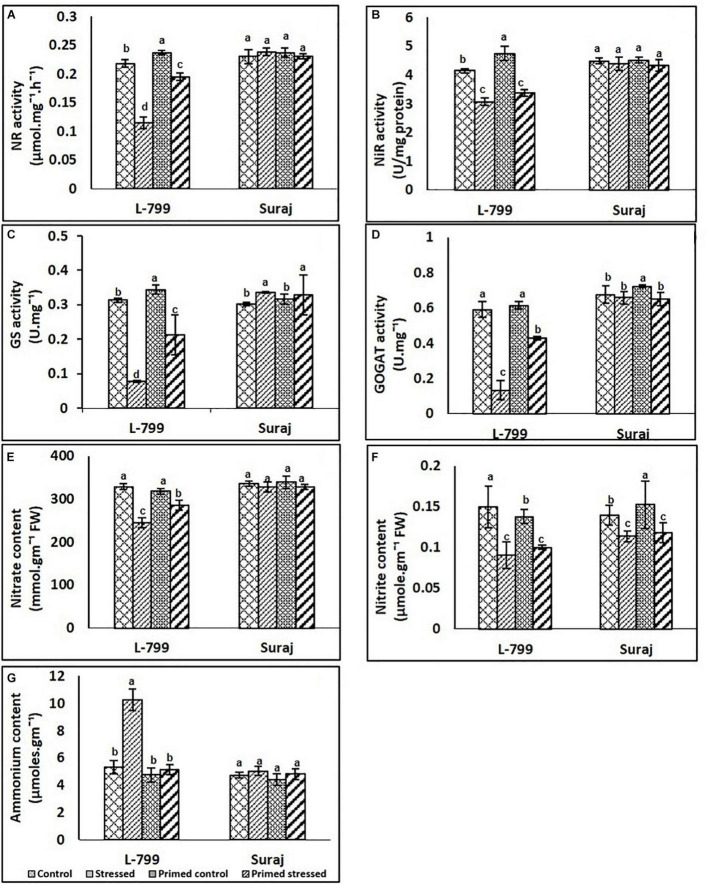
Effects of melatonin priming on the nitrogen cycle **(A–G)** of two drought distinguished cotton varieties, L-799 and Suraj, under drought stress or well-watered conditions. **(A)** Nitrate reductase (NR), **(B)** nitrite reductase (NiR), **(C)** glutamine synthase content (GS), **(D)** glutamate synthase activity (GOGAT), **(E)** nitrate, **(F)** nitrite, and **(G)** ammonium contents. Data represent mean values ± SD of 3 independent experiments. Different alphabets within the group represent significant differences among treatments according to Duncan multiple range test at *P*<0.05.

Melatonin priming augmented the activity of nitrate reductase (NR), nitrite reductase (NiR), glutamate synthase (GS), and glutamine synthase under stress conditions prominently in the L-799, but no change was observed in Suraj (Figure 9). A significant decrease in NR activity was observed in unprimed stressed plants, but priming increased the activity under stress compared to unprimed stressed in L-799. In Suraj, no change was observed across the samples (Figure 9A). No significant change was observed in NiR among unprimed and primed stressed plants of both L-799 and Suraj (Figure 9B). Glutamine synthase (GS) and glutamine synthase (GOGAT) activities decreased significantly under stress compared to control, but priming showcased significantly higher activity than unprimed plants under stress in L-799. In Suraj, both unprimed and primed stressed plants showed similar GS activity, higher than unprimed control (Figures 9C,D). However, the GOGAT activity showed no significant change in unprimed and primed stressed compared to unprimed control in Suraj (Figure 9D).

Nitrate and nitrite content decreased significantly in unprimed stressed plants of L-799 compared to control, but a significant increase was observed in primed stressed plants compared to unprimed stressed plants. In Suraj, no significant change was observed in nitrate content across the samples, whereas nitrite content showed a decrease in both unprimed and primed stressed plants compared to unprimed controls (Figures 9E,F). Drought stress caused a significant increase in ammonium content, whereas primed stressed plants showed no significant change compared to controls in L-799. In Suraj, the ammonia content was the same in all conditions (Figure 9G).

### Effect of Melatonin on the Expression of Stress-Related Genes

**FIGURE 10:**
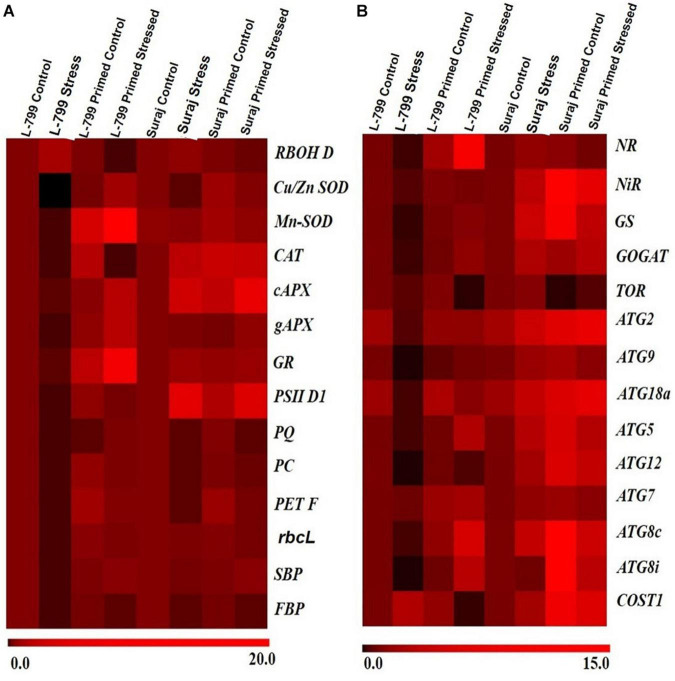
A differential expression pattern of candidate stress-responsive genes in two drought-distinguished cotton varieties, L-799 and Suraj, under drought condition with and without melatonin priming. **(A)** Genes involved in antioxidant systems and photosynthesis, **(B)** genes involved in nitrogen metabolism and autophagy. Values are means of 3 independent experiments.

To support the morphological, biochemical, and physiological data, a set of genes of key enzymes associated with drought-stress response, *viz*. ROS regulation, photosynthesis, nitrogen metabolism, and autophagy was selected for expression profiling (Figures 10A,B). Drought stress increased the *RBOH D* transcript level in unprimed plants by 2.5-fold and 1.4-fold in L-799 and Suraj (Supplementary Figure 5A). Melatonin priming under stress conditions downregulated the expression by 50-fold in L-799 and 3.5-fold in Suraj compared to unprimed stressed plants. The transcript levels of all the antioxidant enzymes (*SOD, CAT, APx*, and *GR*) drastically decreased in unprimed stressed plants of L-799. Melatonin priming significantly elevated the transcripts levels of all antioxidant enzymes compared to unprimed plants under stress in L-799 (Supplementary Figures 5B–G). In the tolerant variety, Suraj, only the *Cu/Zn SOD* level decreased under stress by threefold in unprimed plants. Moreover, the *Mn-SOD* and *gAPX* levels were statistically similar, whereas the *CAT*, *cAPX*, and *GR* levels were elevated 4-, 3-, and 1.75-fold, respectively, under drought stress compared to unprimed controls. Primed stressed plants exhibited similar transcript levels for *Mn-SOD, gAPX*, and *GR* compared to unprimed stressed plants, and *CAT* and *cAPX* increased in primed stressed plants compared to unprimed stressed plants in Suraj (Supplementary Figures 5B–G).

Water stress is well-known for mitigating the photosynthetic efficiency by hampering the electron transport chain, but the primed stressed plants seemed to have better photosynthetic outcomes. The unprimed stressed plants of L-799 showed a significant downfall in transcripts levels related to chloroplastic ETC, *viz*, the level of *PSII D1* (Supplementary Figure 6A), *plastoquinone* (Supplementary Figure 6B), *plastocyanin* (Supplementary Figure 6C), *and ferredoxin* (Supplementary Figure 6D). But primed stressed plants of L-799 exhibited a significant increase in all the transcripts compared to unprimed stressed plants, respectively. The unprimed Suraj stressed plants also displayed a decreased transcript level, but the fold-changes were 11-fold, 2.7-fold, 11-fold, and 4.5-fold, respectively, which were lesser than the unprimed stressed plants of L-799. The primed stressed plants of Suraj showed a significant increase in *PC* and *Fd* transcripts levels, but no significant change in *PSII D1* and *PQ* levels was observed compared to unprimed stressed of Suraj (Supplementary Figures 6A–D). The transcript level of the *RUBISCO (rbsL)*, the first enzyme of the C_3_ cycle, large subunit decreased 8.7-fold and 1.7 fold in the unprimed plants of L-799 and Suraj, respectively, under stress (Supplementary Figure 6E). But the primed stressed plants showed a 8.4-fold higher transcript level in L-799 compared to the unprimed. The transcripts level of the other two important enzymes of C_3_ cycle *FBP* and *SBP* decreased drastically in the L-799 unprimed stressed plants (Supplementary Figures 6F,G). But, in the primed stressed plants, the transcripts level increased significantly compared to the unprimed stressed plants in L-799. The *SBP* transcript level decreased 1.7-fold, and the *FBP* decreased fivefold in the unprimed plants compared to controls in tolerant variety Suraj. Melatonin priming under stress conditions has no significant effect on *FBP* transcripts level, but the *SBP* level increased 1.84-fold compared to the unprimed stressed plants of Suraj (Supplementary Figures 6F,G).

The transcript levels of *NR* (Supplementary Figure 7A), *NiR* (Supplementary Figure 7B), and *GOGAT* (Supplementary Figure 7D) decreased fivefold, 2.3 fold, and 5.73-fold, respectively, in the L-799 stressed plants. But the *GS* (Supplementary Figure 7C) level decreased drastically by 50-fold. On the other hand, in Suraj, the stressed condition upregulated the transcripts level of NR (1.68-fold), *NiR* (3.15-fold), *GOGAT* (2.53-fold), *GS* (4.1-fold) (Supplementary Figures 7A–D). But, in the L-799 primed stressed plants, the transcript levels increased significantly in *NR* (54-fold), *NiR* (2.25-fold), *GS* (65-fold), and *GOGAT* (9-fold) compared to the unprimed stressed plants (Supplementary Figures 7A–D). Melatonin treatment seemed to have no significant effect on the *GS* transcript level, but *NiR* and *GOGAT* levels were upregulated significantly compared to the unprimed stressed plants of Suraj under drought stress.

The genes related to autophagy showed differential expression patterns under stress and primed stressed conditions (Supplementary Figures 8A–J). The level of *TOR* (Supplementary Figure 8A), a negative regulator of autophagy, decreased significantly by 8.7-fold in L-799 and threefold in the Suraj primed stressed plants compared to the unprimed stressed plants of respective varieties. The transcript levels of *ATG2* (Supplementary Figure 8B), *ATG9* (Supplementary Figure 8C), *ATG5* (Supplementary Figure 8E), and *ATG12* (Supplementary Figure 8F) decreased significantly under stress in L-799, but priming treatment elevated the levels compared to the unprimed plants under stress. In Suraj, *ATG2* transcript levels increased, but the *ATG9* and *ATG12* levels decreased significantly under primed stress conditions compared to the unprimed stressed plants. The transcript level of *ATG7* (Supplementary Figure 8G) was significantly reduced in L-799 but increased in Suraj under stress. In primed stressed plants of L-799, the level increased 2.8-folds compared to the unprimed stressed plants, but no significant change was observed in Suraj. *ATG18a* (Supplementary Figure 8D), an important autophagic regulator in plants under water stress, showed a distinct expression pattern in the two varieties. The level decreased drastically under the stressed L-799 plants, but in Suraj, the expression level increased 3.8-fold in the unprimed stressed plants compared to controls. A significant rise in the expression level of *ATG18a* was exhibited in both varieties in the primed stressed plants compared to the unprimed stressed plants. The two *ATG8* orthologs, i.e., *ATG8c* (Supplementary Figure 8H) and *ATG8i* (Supplementary Figure 8I), showed similar expression patterns in both varieties. In L-799, the stressed plants showed decreased expression levels by 3.5-fold and 5.5-fold compared to controls, respectively. Priming increased the expression level significantly compared to the unprimed plants under stress conditions. In Suraj, the transcript levels of *ATG8c* (Supplementary Figure 8H) were elevated 3.8-fold, but *ATG8i* showed no significant difference compared to controls in stressed plants. The primed stressed plants of Suraj exhibited a significant increase in the ATG8i transcript level but similar ATG8c levels compared to the unprimed stressed plants. *COST1* (Supplementary Figure 8J) is a negative regulator of autophagy but has a positive regulatory function in plant development. The transcript level of *COST1* increased 2.7-fold in L-799 and 2.24-fold in Suraj during stress in the unprimed plants compared to controls. The primed plants showed decreased *COST1* by 21-fold in L-799, but it increased 2.5-fold under stress in Suraj compared to respective unprimed stressed plants.

### Expression Level of Non-lipidated (Free ATG8) and Lipidated (ATG8-PE) Indicators of Autophagy

**FIGURE 11:**
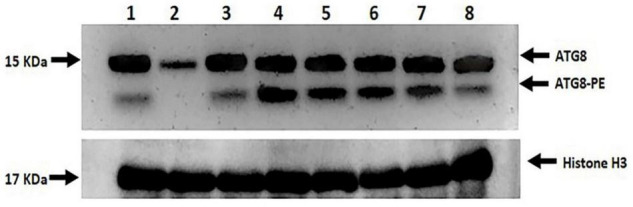
Immunoblot analysis of ATG8 and ATG8-PE in melatonin primed and unprimed drought-sensitive (L-799) and -tolerant (Suraj) varieties under drought stress or optimal conditions. Lane 1- L-799, control; Lane 2- L-799, stressed; Lane 3- L-799, primed control; Lane 4- L-799, primed stressed; Lane 5- Suraj, control; Lane 6- Suraj, stressed; Lane 7- Suraj, primed control; and Lane 8- Suraj, primed stressed.

Two prominent bands, ATG8 (upper band, free ATG8) and ATG8-PE (lower band, ATG8-PE), were found between 15 and 10 KDa, which were highly modulated by both drought stress and melatonin (Figure 11). No or undetectable expression level of ATG8-PE and the relatively lower ATG8 expression level was observed in L-799 stressed plants. In contrast, a relatively higher ATG8-PE expression was detected in the primed stressed plants compared to controls. In Suraj, the ATG8 expression level was similar in control and stressed conditions, whereas the ATG8-PE level was relatively higher in the stressed plants. Surprisingly, the expression level of ATG8-PE was decreased in Suraj primed stressed compared to the unprimed stressed and control plants (Figure 11).

## Discussion

Priming of seeds with different molecules to achieve tolerance against various abiotic and biotic stresses is a well-known practice (Paparella et al., 2015). One such chemical is melatonin, which has a profound effect on ROS homeostasis, plant growth and development, germination, and photosynthetic efficiency. Thus, melatonin is emerging as an answer for withstanding several environmental stress factors in diverse crops and plant species. It also cross-talks with stress-related phytohormones and modulates the expression patterns of genes and transcription factors related to stress (Arnao and Hernández-Ruiz, 2014, 2019). Two upland cotton varieties, distinguished by drought-stress tolerance, were chosen to understand the differential effect of melatonin under drought conditions. The drought-sensitive variety, L-799, showed severe wilting symptoms and stunted growth, but the drought-tolerant variety, Suraj, exhibited a normal phenotype with no wilting under drought stress. Melatonin (10 μM) priming implicated a positive phenotypical effect on L-799 stressed plants that make them morphologically indistinguishable compared to the control plants. But Suraj did not display any notable morphological effect of melatonin priming under the drought-stress condition (Figure 1 and Supplementary Figure 1).

### Exogenous Priming Caused an Endogenous Increment in Melatonin Content

The melatonin level counters the stress-induced adverse effects in the plants, as the plants with higher content were found to have better stress tolerance (Bajwa et al., 2014; Antoniou et al., 2017). In this study, decreased melatonin content under stress and elevated levels in the primed stressed plants of L-799 suggests its involvement in stress tolerance. Similar melatonin levels in both the unprimed plants and the primed plants under stress conditions in the Suraj variety are in accordance with Suraj’s inherent stress-tolerance nature. The exogenous priming had no further beneficial effect on drought tolerance (Figure 4). Moreover, a non-significant decrease in melatonin content in the primed stressed plants compared to the unprimed stressed plants of Suraj in this study indicates the feedback inhibition of melatonin on its own biosynthesis (Back et al., 2016) due to higher content.

### Priming Alleviated the Oxidative Stress Under Drought Stress by Regulating the Enzymatic and Non-enzymatic Antioxidant Systems

Drought stress has been reported to increase the oxidative stress level in the cell (Mattos and Moretti, 2015) by increasing the ROS production through upregulation of the RBOH D expression (Suzuki et al., 2011) and degradation of the ROS-detoxification system (Ren et al., 2016). Other reports also stated the regulatory role of melatonin in the mitigation of superoxide and hydrogen peroxide levels (Huang et al., 2019) and elevation of antioxidant systems (Li C. et al., 2015). Here, significantly higher superoxide radicals and H_2_O_2_ levels signify the unfavorable condition of L-799 under stress, but priming justifies its role by mitigating both levels. However, in Suraj, equivalent levels of both superoxide radicals and H_2_O_2_ in the stressed and primed-stressed plants are an add-on over the phenotypical observation (Figure 2). The higher transcript level of *RBOH D* under stress in both varieties, and notably, in L-799 (Figure 10A), justifies the higher superoxide radical content. Elevated H_2_O_2_ with lowered Cu/Zn-SOD and Mn-SOD transcript levels indicates either spontaneous H_2_O_2_ generation or the failed downstream processes of quenching H_2_O_2_ in the L-799-stressed plants (Figures 2D, 10A). But relatively higher transcript levels of *Cu/Zn-SOD*, *Mn-SOD*, and decreased *RBOH D*, superoxide radicals, and H_2_O_2_ in the primed stressed plants of L-799 compared to Suraj are pointing toward the exclusive effectiveness of melatonin in a sensitive variety only. L-799 showed a decrease in CAT activity and a transcripts level under stress (Figure 3B), indicating higher H_2_O_2_ content but priming caused upregulation in both the activity and the transcript level, justifying the lowered H_2_O_2_. Contrastingly, drought showed no negative impact on GPoX activity in both the varieties under stress, although priming significantly upregulated the activity in L-799 (Figure 3C). The ascorbate-glutathione cycle is a ROS-scavenging pathway that helps mitigate oxidative conditions inside the cell and maintain cellular harmony, where drought stress creates an unbalanced state (Guo et al., 2020). Diminution in ascorbate and a reduced glutathione level along with MDAR and DHAR activity with an increased oxidized glutathione level in L-799 stressed plants (Figure 5) points toward an unbalanced AsA-GSH cycle. The opposite scenario in the Suraj-stressed plants indicates a maintained AsA-GSH cycle. Melatonin priming upregulates the ascorbate, GSH content, and MDAR and DHAR activity, and decreases oxidized glutathione content in L-799 under stress, thus maintaining the AsA-GSH pool under drought stress, which corroborate the findings in maize and wheat (Guo et al., 2020). On the other hand, priming did not show any significant effect on the AsA-GSH cycle, possibly due to its tolerant nature, which is in accordance with the earlier report in wheat (Loggini et al., 1999). The ratio of GSH and GSSG is another grandiloquent factor as it indicates the stress level, and melatonin elevates the ratio to provide better stress tolerance (Bidabadi et al., 2020). In this study, the lowered GSH and higher GSSG in unprimed plants but elevated GSH and decreased GSSG contents in primed plants justifies the melatonin’s effect on drought stress mitigation in L-799 under stress. In Suraj, the egalitarian ratio, irrespective of condition and priming, indicates its inherent stress tolerance capacity, where melatonin was found to be ineffective.

### Melatonin Enhances the Photosynthetic Parameters Under Drought Stress

Drought stress has been reported to negatively impact photosynthesis due to decreased chlorophyll content (Wang et al., 2018; Xu et al., 2020), stimulated stomatal closure followed by a decline in stomatal conductance and a transpiration rate (Farooq et al., 2009). Additionally, decreased transpiration leads to *RUBISCO* oxygenation followed by photorespiration, creating limitations in biomass production, and higher ROS production. Here, the decline in the photosynthesis rate, stomatal conductance, and transpiration rate under stress, predominantly in L-799, correlates with the decreased chlorophyll level (Figure 7), which is linked to an increased transcripts level of *PAO*, a chlorophyll degrader (Supplementary Figure 4). In the primed stressed plants of L-799, the mitigated level of *PAO* with higher chlorophyll content justified the higher photosynthesis rate along with stomatal conductance and the transpiration rate as reported by Zhao et al. (2017). Here, unchanged photosynthetic rate, chlorophyll content, and *PAO* transcripts level in both primed and unprimed stressed plants of Suraj proves that melatonin has no additional beneficial effect in tolerant variety. The decreased total soluble sugar and transcript levels of *rbcsL*, *FBP*, and *SBP* genes under drought in L-799 (Figure 10) indicate a decrease in C_3_-cycle activity, which might be the cause of lower biomass and lesser growth, which was inversed in both the varieties in the primed stressed plants pointing toward the role of melatonin in positive regulation of C_3_. Similar to the observation in maize (Huang et al., 2019), priming increased the WUE of both varieties in this study (Figure 7). However, the significant change in L-799 plants depicts the better effect of melatonin priming under drought stress.

Photosynthetic efficiency is an effective way to measure the physiological condition of the plants under drought as it has a detrimental effect on F_v_/F_m_ (Meng et al., 2014). Drought condition also creates hindrance in quantum yield of PS (II) and ETR (II) (Wang et al., 2018). In this report, a significant decrease in the F_v_/F_m_ level in unprimed and increased Fv/Fm in the primed plants under stress in L-799 and unaltered F_v_/F_m_ in Suraj under all conditions (Figure 8) indicates that the shielding effect of melatonin is biased toward L-799. The quantum yield of PS (II) and ETR (II) was also severely decreased in L-799, which could be a result of fatally damaged PS (II). The decrease in photosynthesis might have been due to the absorption of more light energy than needed, resulting in higher ROS generation and hindering D1 protein synthesis (Takahashi and Murata, 2008). Thus, decreased *PS* (*II*) *D1* transcripts and ETR (II) are linked by cause-effect relationship. In contrast, higher PS (II) D1 indicates the protective role of melatonin in D1 protein in the primed plants, as reported by Huang et al. (2019) in maize under drought. Antoniou et al. (2017) reported an increase in the transcripts level of genes related to electron transport under drought stress in the melatonin primed plants of *Medicago sativa*. In this study, significant elevation in transcripts levels of *PQ*, *PC*, and *PET F* in L-799 under primed stressed conditions indicates the preference of melatonin toward L-799 (Figure 10). NPQ is a protective mechanism by which a plant quenches excess-absorbed light energy into heat and reduces the photodamage (Zhao et al., 2017). Priming elevated the NPQ level in L-799, which stands for better energy quenching efficiency, but no additional benefit was observed in Suraj under stress (Figure 8). The Y (NO) and Y (NPQ) define the difference in the energy dissipation process between the two varieties, *viz.* increased Y (NO) reflects a restricted proton gradient across thylakoid membrane (Perreault et al., 2009; Huang et al., 2012). Thus, the increase in the Y (NO) level in L-799 under stress might be the result of a restricted proton gradient, which is another indication of damaged PS (II). Moreover, the decreased Y (NPQ) level dictates either a lower activity of the xanthophyll cycle (Khan et al., 2017) due to a slow rate of energy dissipation (Epron and Dreyer, 1992) or higher membrane damage that reduced the proton gradient as found in L-799 under the stress condition. But, in Suraj, increased Y (NPQ) lowers the pressure of energy dissipation for PS (II) under drought. Similar to the report of Guo et al. (2020) in maize, melatonin has affected Y (NO) mitigation and Y (NPQ) elevation in both the varieties, significantly in sensitive L-799, indicating that melatonin might involve in balancing the pH-gradient across the thylakoid membrane and maintaining the membrane structure.

### Melatonin Ameliorates Lipid Peroxidation by Increasing the α-Tocopherol Content Under Drought

Electrolytic leakage indicates membrane damage, which is a common detrimental effect of drought stress, with the primary cause being lipid peroxidation (Cherono et al., 2020; Sadak et al., 2020). Here, elevated MDA and electrolytic leakage levels indicate the pernicious effect of drought stress in L-799, but the non-significant changes in Suraj suggest its stress-tolerance nature (Figure 6). α-tocopherol has the potential to scavenge superoxide and peroxyl radicals and convert them into tocopherol radicals, which are recycled back by ascorbate (Munné-Bosch, 2005). In this study, priming significantly upregulated α-tocopherol content in the stressed plants of both varieties. This could be due to the positive regulatory role of melatonin or the increased ascorbate levels in the varieties under stressed conditions.

### Priming Resulted in Better Nitrogen Metabolism by Upregulating the Transcript Levels With a Concomitant Increase in Enzyme Activity of Related Genes

The nitrogen cycle is an essential phenomenon that deals with plant biomass production, growth, and development. Nitrate is one of the dominant forms of nitrogen that plants take up from the soil (Qiao et al., 2019). Decreased nitrate and nitrite contents and related enzyme activities and the transcript level of genes under stress indicate lower uptake and conversion of nitrate in L-799 under stress. However, increased NR activity (Figure 9) and NR transcripts (Figure 10) level indicates higher nitrate uptake and better conversion of nitrate to nitrite, thus preventing over-accumulation of nitrate and better nitrogen metabolism in the primed stressed plants of sensitive variety (L-799). For Suraj, no change in the NR activity, nitrate content, and the increased *NR* transcript level under stress delineates the stress tolerance capacity and better nitrate uptake and conversion, on which melatonin priming has no further beneficial effect. Contrary to the report in maize (Erdal and Turk, 2016), melatonin priming had no effect on nitrite content as the unprimed and primed plants exhibited similar nitrite content, NiR activity, and a *NiR* transcript level in L-799 under stress. Ammonium is an inorganic nitrogenous compound, which in a higher amount, creates cellular toxicity (Erdal and Turk, 2016; Ashraf et al., 2018). Higher ammonium content in unprimed plants but lower content in primed plants showcases the melatonin’s effectiveness against ammonium accumulation in L-799 under stress, which corroborates the findings in melon under sub-low temperature stress (Gao et al., 2016). Generally, in the case of higher plants, the assimilation of ammonium into organic compounds occurs by a GS/GOGAT cycle, where ammonium is first converted to glutamine by the activity of glutamine synthase and then from glutamine to glutamate by the activity of GOGAT (Du et al., 2017; Ashraf et al., 2018). The mitigated GS and GOGAT activity and transcript levels in unprimed stressed plants and upregulation in primed stressed plants justify the priming effect against ammonium accumulation by converting it into glutamine, glutamate, and better production of N_2_-compounds for improved growth and development in L-799. Higher enzyme activities and better nitrate and nitrite contents with decreased ammonium content under drought stress than L-799 indicated better nitrogen metabolism in Suraj. Surprisingly, no change observed in the content and activities of the enzymes related to the nitrogen cycle among stressed and primed stressed plants of Suraj justifies the ineffectiveness of melatonin in it (Figures 9, 10).

### Melatonin Priming Has a Positive Regulatory Function in Autophagy Induction Under Drought Stress Conditions

**FIGURE 12:**
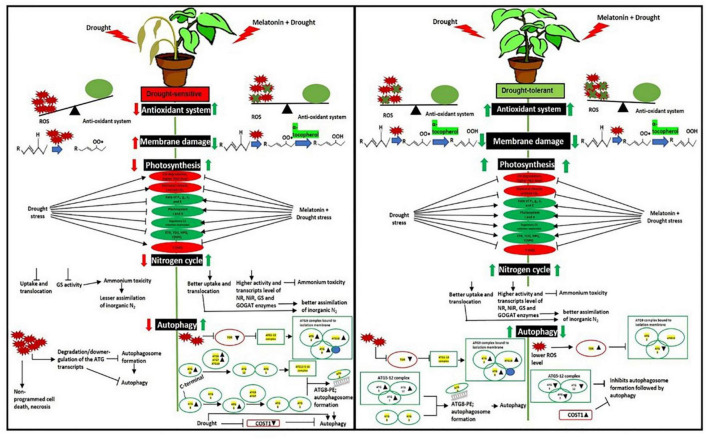
Schematic representation of mechanistic differences of the effects of melatonin priming on drought-tolerant (L-799) and drought-sensitive (Suraj) varieties of upland cotton under drought stress. Improved antioxidant systems, photosynthetic machineries, and nitrogen metabolism substantiate the positive effect of melatonin on L-799 under drought stress. Exogenous melatonin priming did not provide any further beneficial effect on drought tolerant variety possibly due to the higher endogenous melatonin content, which is in itself sufficient for drought tolerance and thus could be turning off the effects of exogenous melatonin due to feedback inhibition. Melatonin priming was found to differentially regulate ROS levels and thus autophagy in drought-sensitive and -tolerant varieties.

Drought-induced ROS can also cause protein oxidation and the malfunctioning of metabolic processes that may induce autophagy (Pérez-Pérez et al., 2012), which is essential for better tolerance (Liu et al., 2009). The initiation starts with the stress signal perceived by TOR kinase, a negative autophagy regulator (Fu et al., 2020). In both L-799 and Suraj, a significantly lower *TOR* level in primed plants than unprimed under stress indicates the melatonin involvement for better autophagic induction under stress. TOR also has a positive role in vegetative growth and development in plants (Rexin et al., 2015), which might be the reason for a higher transcripts level in Suraj unprimed stressed plants, or could be the consequence of post-transcriptional modifications (Figure 10). *ATG18* is another gene that helps in the formation of autophagosomes (Obara et al., 2008; Watanabe et al., 2012) along with ATG2 (Wang et al., 2001) and ATG9 (Watanabe et al., 2012; Zhuang et al., 2017). A significant decrease of *ATG2*, *ATG18a*, and *ATG9* in L-799 but an increase in Suraj variety shows the differential gene expression and regulation under drought in two varieties. A significantly higher transcripts level of these genes in primed stressed plants of both varieties compared to respective unprimed control plants indicates the effectiveness of melatonin in autophagosome formation. ATG8-PE, the ultimate form of autophagosome formation, requires the ATG5-ATG12-ATG16 complex (Noda et al., 2013). A significantly lower abundance of *ATG5* and *ATG12* transcripts under drought in L-799 indicates the inertia in autophagic activity, but the elevated level in primed stressed plants suggests the effectiveness of priming. In Suraj, the already increased transcripts level in unprimed stress plants indicates that Suraj can maintain autophagic activity without melatonin, unlike L-799. ATG7 is an E1-like enzyme that transfers the ATG3, an E2-like enzyme, to ATG8 (Han et al., 2011). Although the transcripts level of *ATG7* was similar both in control and stress in L-799, the significant decrease of *ATG8c* and *ATG8i* transcripts suggest the negative impact of drought on ATG8-PE formation. But the elevated transcripts level under drought in primed plants justifies the positive effect of melatonin for autophagy induction under drought. Increased *ATG7*, *ATG8c*, and maintained *ATG8i* transcripts levels under stress in unprimed Suraj indicate its instinctive autophagic induction without melatonin treatment. Another noteworthy finding is that, in the primed plants of Suraj, the relative abundance of *ATG9* and *ATG12* transcripts decreased compared to unprimed plants under stress, which can be explained by a direct effect of melatonin in ROS scavenging. Possibly due to a lower ROS level under melatonin treatment (in Suraj), the autophagic induction was minimized in the primed stressed plants, as a higher ROS level induces autophagy, but melatonin inhibits the ROS (Zhao et al., 2021). COST1 protein renders the formation of ATG8-PE, thus inhibiting autophagy under normal conditions, but it has a pivotal role in the growth and development of the plant, guided mainly by external conditions (especially drought) (Bao and Bassham, 2020; Bao et al., 2020). An increased *COST1* transcript level under drought showcases the chances of abated autophagic activity in L-799, which may be correlated with the lower transcript level of *ATG8* (*ATG8c* and *ATG8i*). Furthermore, the absence of ATG8-PE expression clarifies the above mentioned observation. A significant decrease in *COST1* expression in primed stressed plants with a higher expression of ATG8-PE indicates the better autophagic induction under stress, which might be an effect of priming. The higher expression level of *COST1* and lower ATG8-PE expression in the primed stressed plants compared to the stressed plants, unlike L-799, justifies the vigorous phenotype and lower autophagy inductive effect of melatonin in Suraj under stress (Figures 10, 11). Thus, the differential regulation of the autophagy process in drought-sensitive and -tolerant varieties could result from differential modulation of ROS levels by melatonin under drought stress. The proposed model showing the melatonin-mediated drought tolerance in sensitive and tolerant varieties of cotton based on the results obtained in the study is presented in Figure 12.

In summary, melatonin seed priming imparted drought tolerance in drought-sensitive variety (L-799) of cotton by enhancing the antioxidant system and nitrogen metabolism, which could have improved the plant growth and development under drought stress conditions. Increased photosynthetic efficiency with higher chlorophyll content justifies better light energy utilization and food production in melatonin primed stressed plants. In addition, higher ATG8-PE expression in the primed plants led to better regulation of autophagy, thus justifying better intra-cellular nutrient utilization and management under stress in primed plants of L-799. But, in case of Suraj, a drought-tolerant variety, the differences between unprimed and primed for antioxidant systems activity, photosynthetic characteristics, nitrogen metabolism, and autophagy were nearly similar under drought, unlike in L-799.

## Conclusion

This study provided insights into the differential regulation of melatonin-induced drought tolerance in drought-sensitive and -tolerant varieties incarnated with antioxidant systems, photosynthesis, nitrogen metabolism, and autophagy. The action of melatonin was found to be variety specific with significant improvement in drought-tolerance response observed in sensitive variety, which was associated with an increase in the antioxidant, improved photosynthetic efficiency and nitrogen metabolism, and autophagy induction, pointing toward their involvement in drought tolerance. The higher endogenous melatonin content in drought tolerant variety could have contributed to its inherent tolerance nature, and exogenous priming had no further beneficial effect. The results also showed differential regulation of autophagy by melatonin in two varieties under drought stress, further substantiating variety-specific effects. Thus, for the first time, the study reported the mechanistic differences of melatonin-induced drought tolerance in drought-sensitive and -tolerant varieties, with distinct beneficial effects conferred in sensitive variety. It would be interesting to evaluate the effect of melatonin in alleviating drought stress in cotton under field conditions at different stages for achieving higher yields in dryland agriculture.

## Data Availability Statement

The original contributions presented in the study are included in the article/Supplementary Material, further inquiries can be directed to the corresponding author/s.

## Author Contributions

GP and LS contributed to conceptualization and experiments design. LS conducted the experiments, data collection, graph preparation, analysis and interpretation of the data, and wrote the manuscript. PD performed few experiments and helped in graph preparation. GP and MM analyzed and interpreted the results and corrected the manuscript. All authors contributed to the work and edited the manuscript.

## Conflict of Interest

The authors declare that the research was conducted in the absence of any commercial or financial relationships that could be construed as a potential conflict of interest.

## Publisher’s Note

All claims expressed in this article are solely those of the authors and do not necessarily represent those of their affiliated organizations, or those of the publisher, the editors and the reviewers. Any product that may be evaluated in this article, or claim that may be made by its manufacturer, is not guaranteed or endorsed by the publisher.

## Footnotes

1
https://www.genscript.com/tools/real-time-pcr-taqman-primer-design-tool
