# Supplementary material for: Melatonin Mediated Differential Regulation of Drought Tolerance in Sensitive and Tolerant Varieties of Upland Cotton (Gossypium hirsutum L.)
Source: Front Plant Sci. 2022 Apr 4;13:821353. doi: 10.3389/fpls.2022.821353 (PMC9014207; doi:10.3389/fpls.2022.821353)
Supplement: Supplementary file 1 [file Presentation_1.PPTX]

## Slide 1
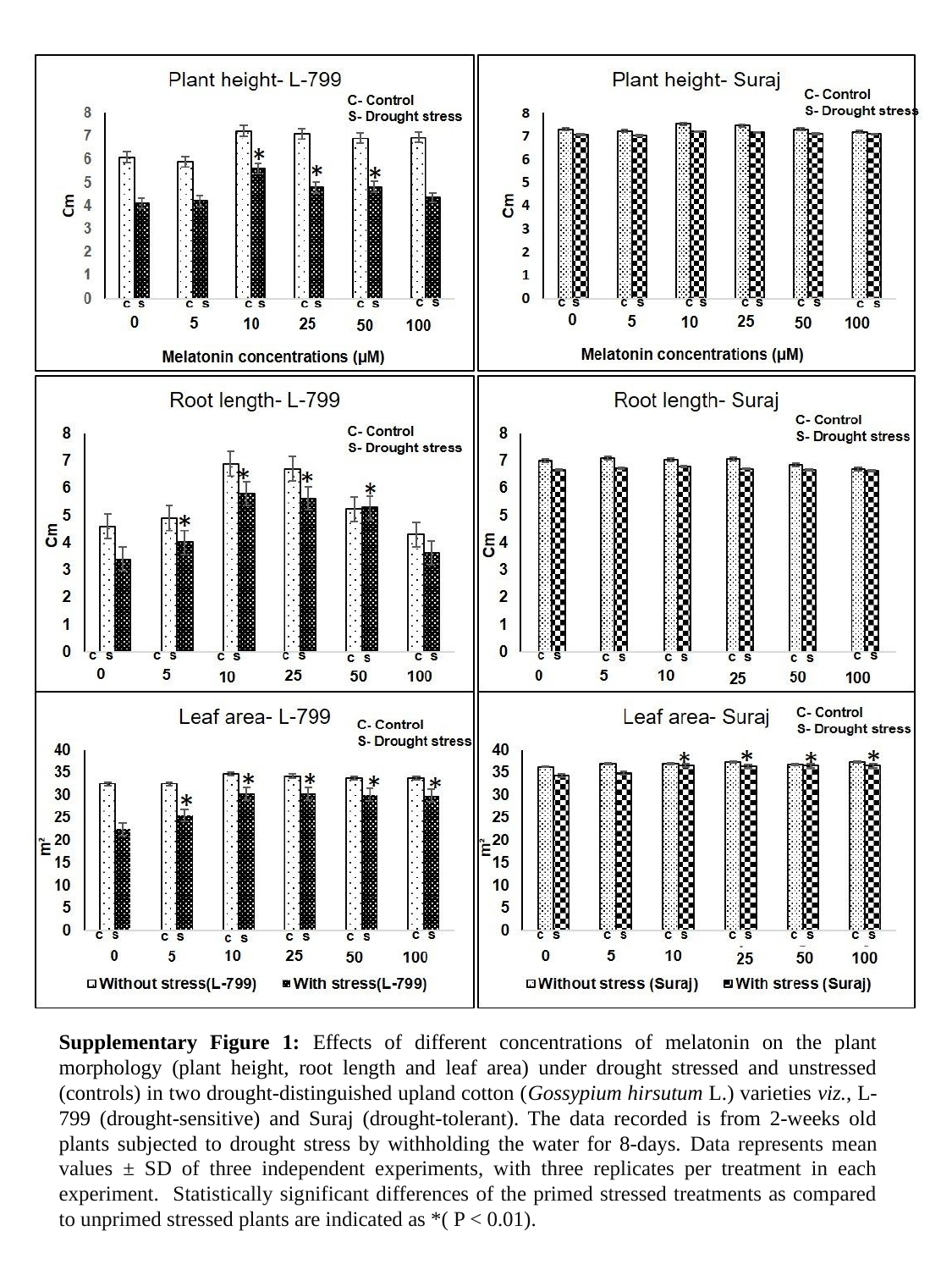

Supplementary Figure 1: Effects of different concentrations of melatonin on the plant morphology (plant height, root length and leaf area) under drought stressed and unstressed (controls) in two drought-distinguished upland cotton (Gossypium hirsutum L.) varieties viz., L-799 (drought-sensitive) and Suraj (drought-tolerant). The data recorded is from 2-weeks old plants subjected to drought stress by withholding the water for 8-days. Data represents mean values ± SD of three independent experiments, with three replicates per treatment in each experiment. Statistically significant differences of the primed stressed treatments as compared to unprimed stressed plants are indicated as *( P < 0.01).

## Slide 2
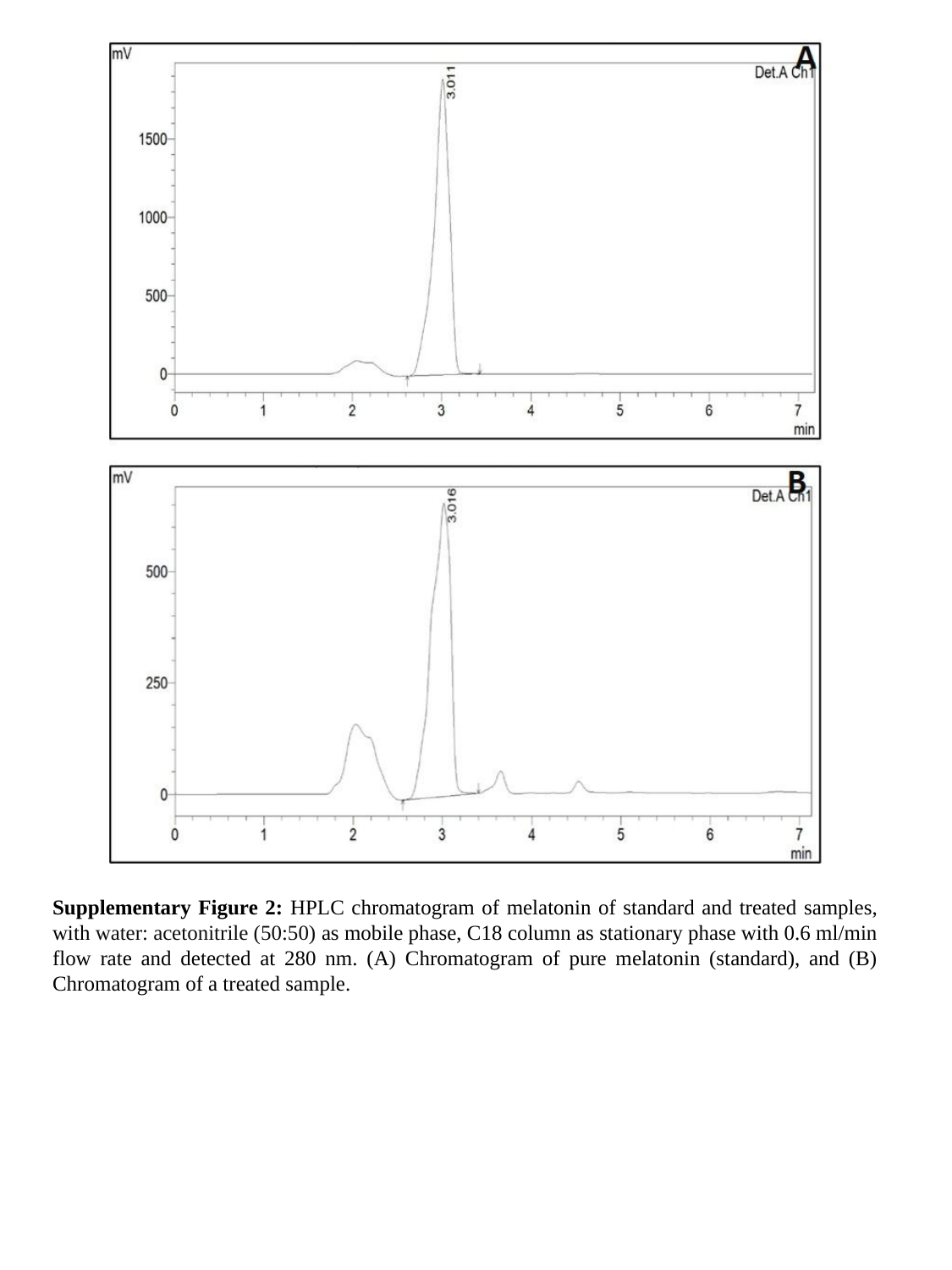

Supplementary Figure 2: HPLC chromatogram of melatonin of standard and treated samples, with water: acetonitrile (50:50) as mobile phase, C18 column as stationary phase with 0.6 ml/min flow rate and detected at 280 nm. (A) Chromatogram of pure melatonin (standard), and (B) Chromatogram of a treated sample.

## Slide 3
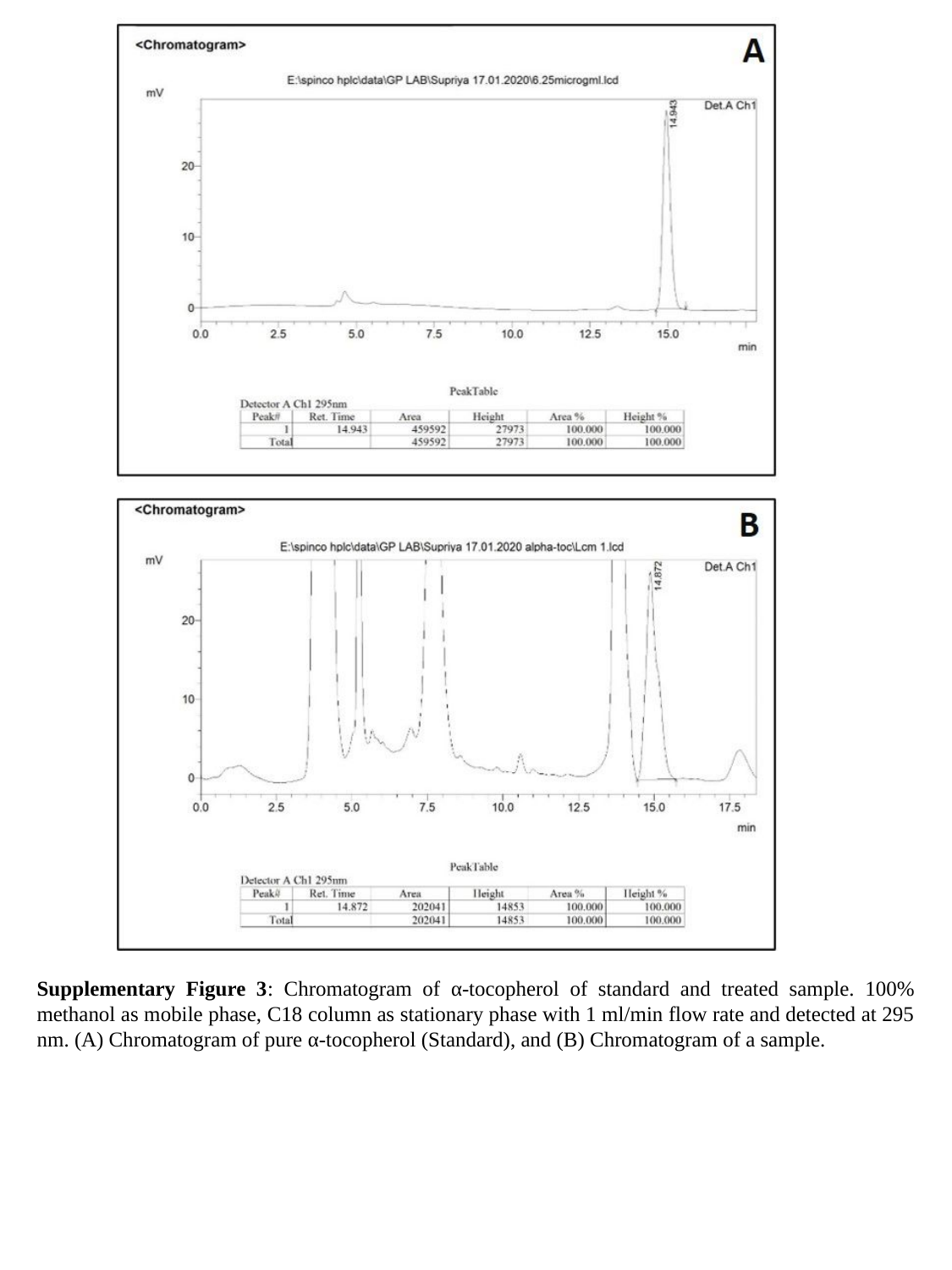

Supplementary Figure 3: Chromatogram of α-tocopherol of standard and treated sample. 100% methanol as mobile phase, C18 column as stationary phase with 1 ml/min flow rate and detected at 295 nm. (A) Chromatogram of pure α-tocopherol (Standard), and (B) Chromatogram of a sample.

## Slide 4
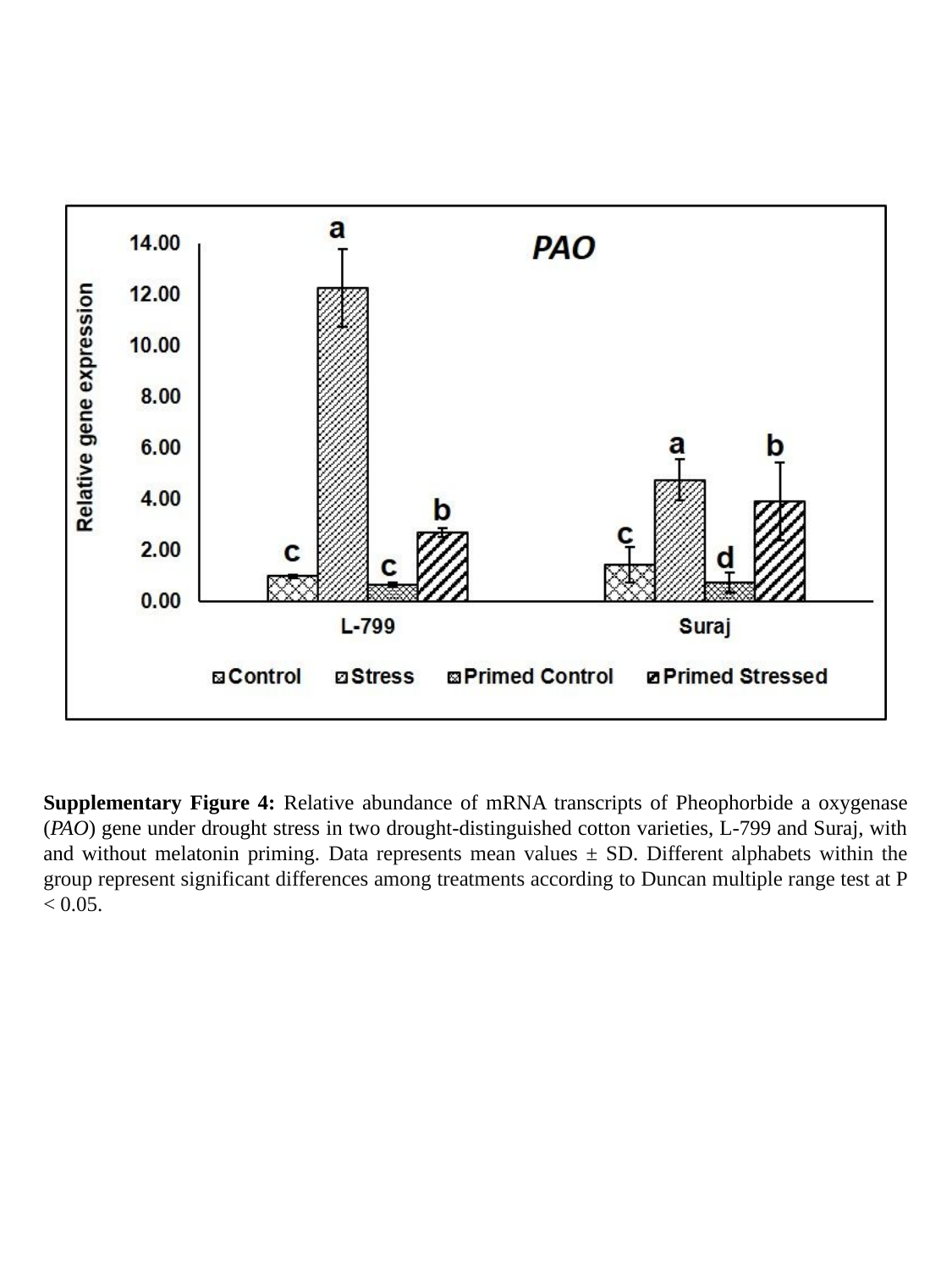

Supplementary Figure 4: Relative abundance of mRNA transcripts of Pheophorbide a oxygenase (PAO) gene under drought stress in two drought-distinguished cotton varieties, L-799 and Suraj, with and without melatonin priming. Data represents mean values ± SD. Different alphabets within the group represent significant differences among treatments according to Duncan multiple range test at P ˂ 0.05.

## Slide 5
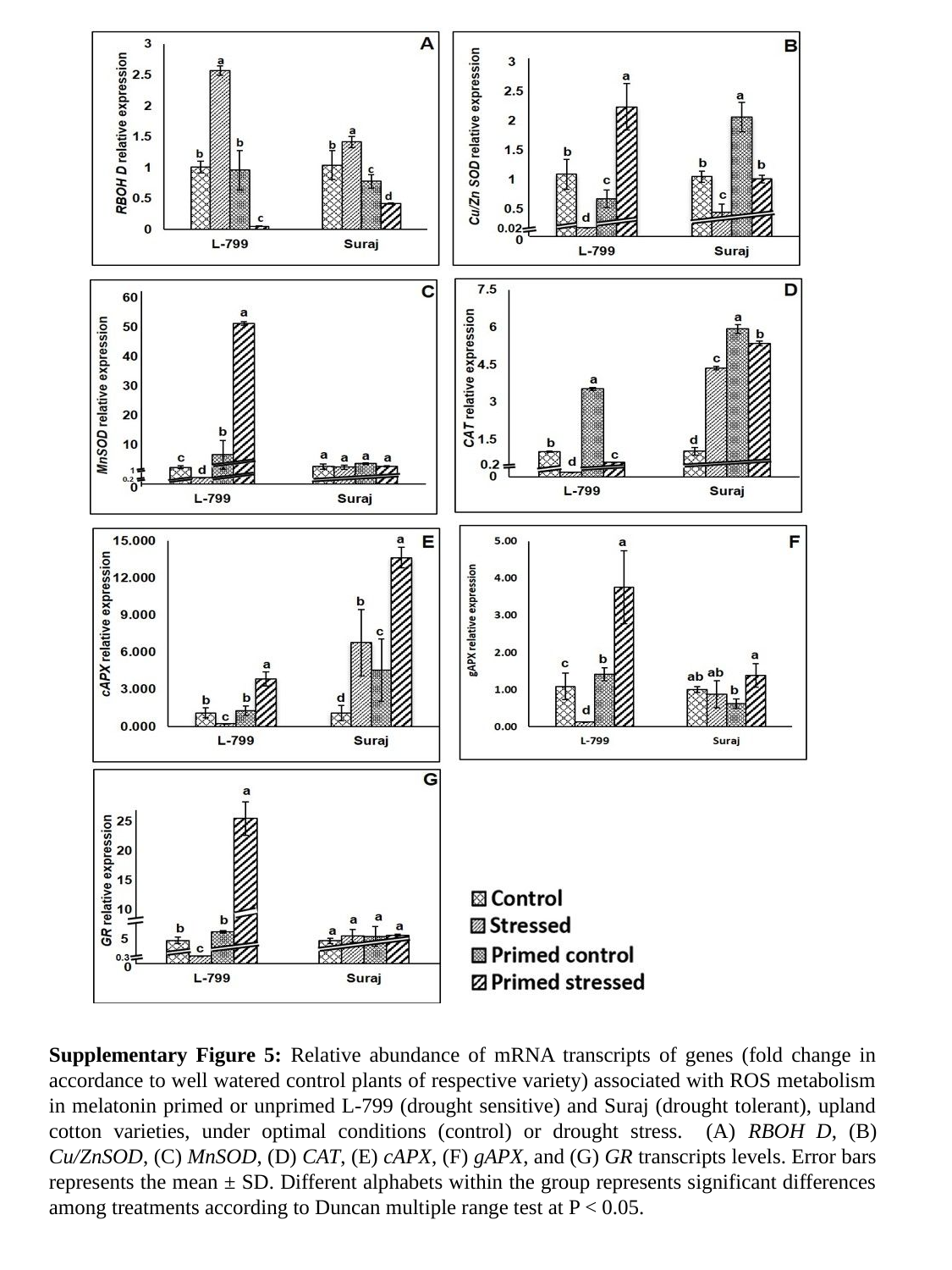

Supplementary Figure 5: Relative abundance of mRNA transcripts of genes (fold change in accordance to well watered control plants of respective variety) associated with ROS metabolism in melatonin primed or unprimed L-799 (drought sensitive) and Suraj (drought tolerant), upland cotton varieties, under optimal conditions (control) or drought stress. (A) RBOH D, (B) Cu/ZnSOD, (C) MnSOD, (D) CAT, (E) cAPX, (F) gAPX, and (G) GR transcripts levels. Error bars represents the mean ± SD. Different alphabets within the group represents significant differences among treatments according to Duncan multiple range test at P ˂ 0.05.

## Slide 6
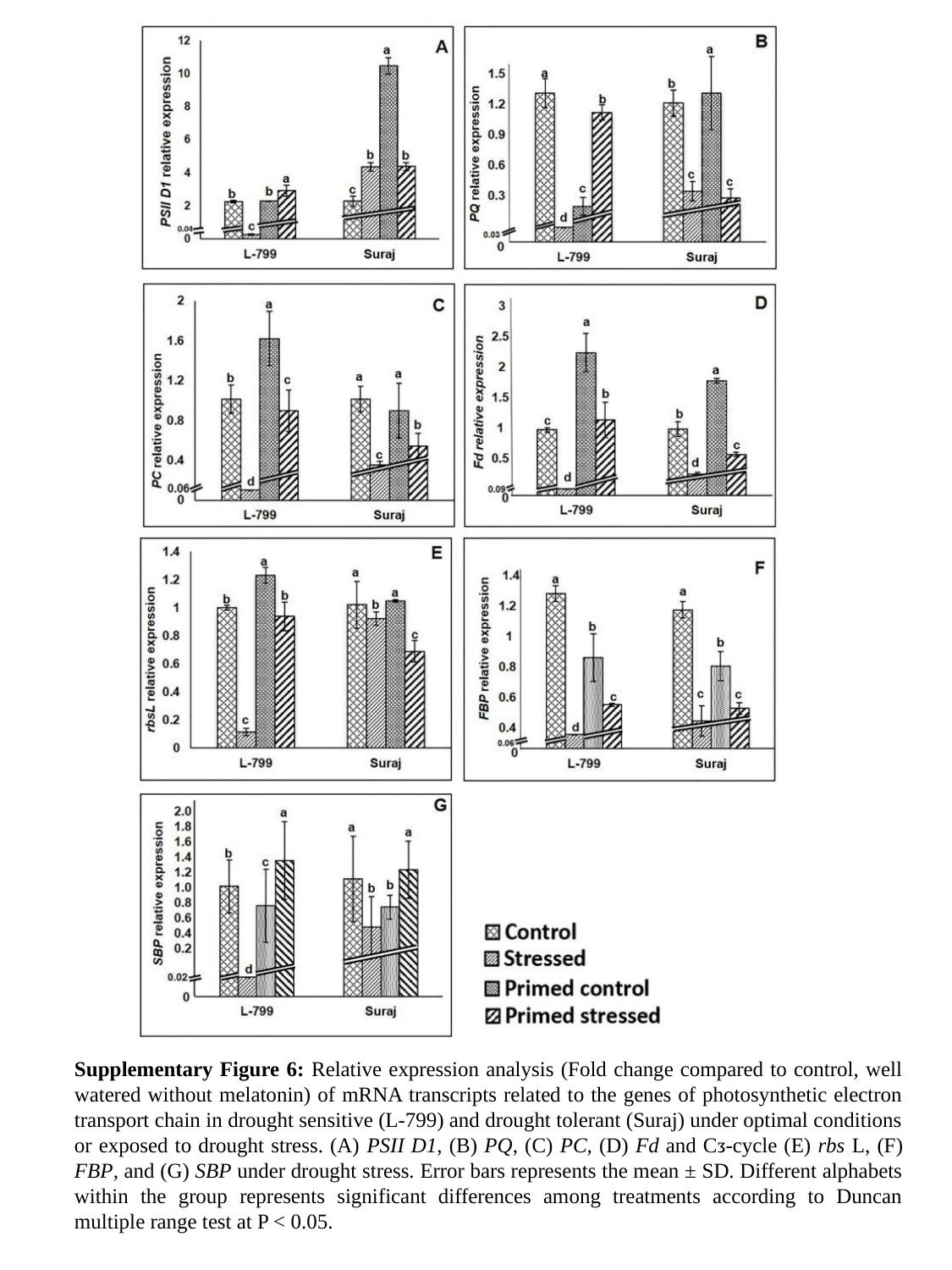

Supplementary Figure 6: Relative expression analysis (Fold change compared to control, well watered without melatonin) of mRNA transcripts related to the genes of photosynthetic electron transport chain in drought sensitive (L-799) and drought tolerant (Suraj) under optimal conditions or exposed to drought stress. (A) PSII D1, (B) PQ, (C) PC, (D) Fd and Cᴣ-cycle (E) rbs L, (F) FBP, and (G) SBP under drought stress. Error bars represents the mean ± SD. Different alphabets within the group represents significant differences among treatments according to Duncan multiple range test at P ˂ 0.05.

## Slide 7
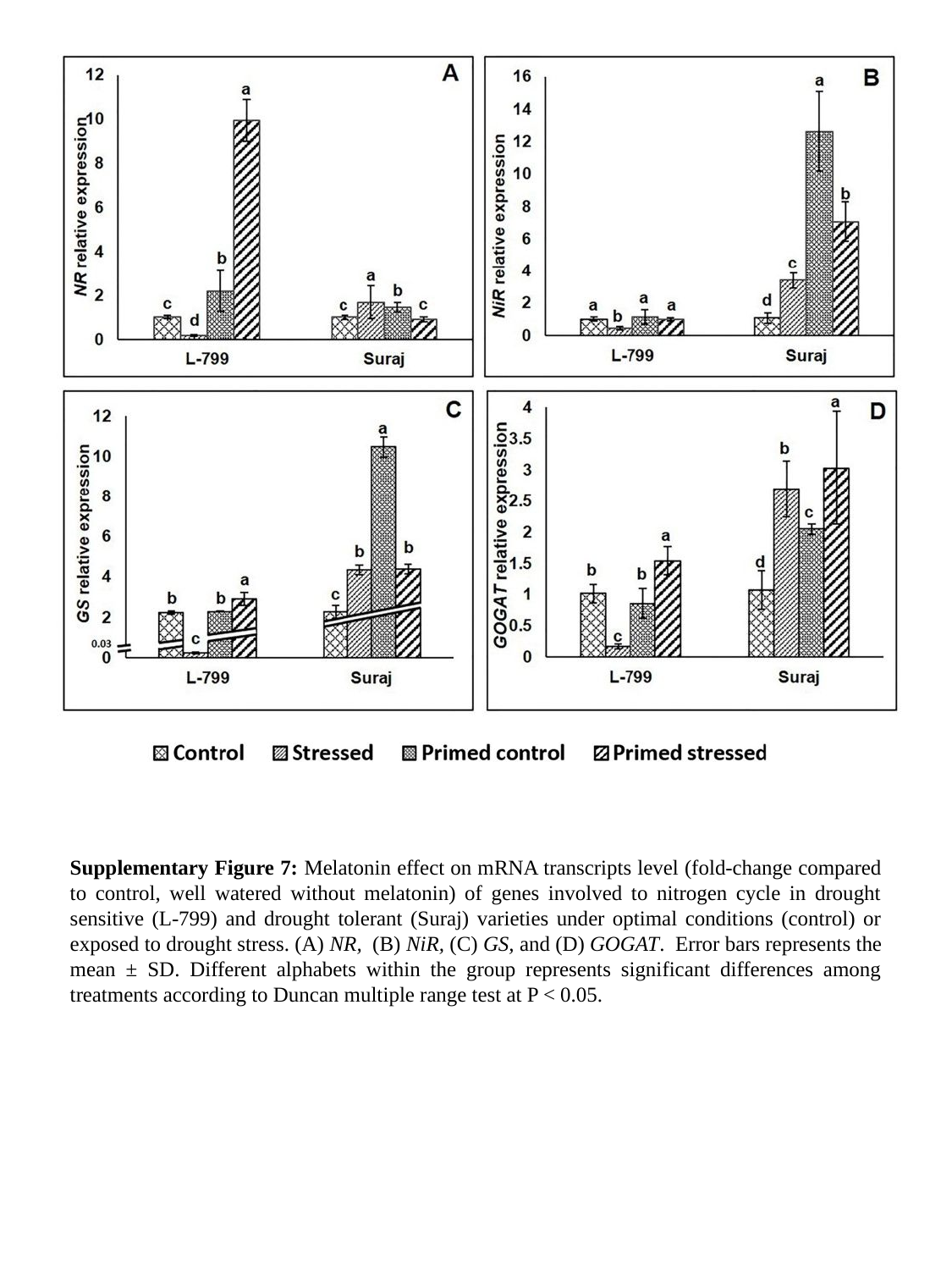

Supplementary Figure 7: Melatonin effect on mRNA transcripts level (fold-change compared to control, well watered without melatonin) of genes involved to nitrogen cycle in drought sensitive (L-799) and drought tolerant (Suraj) varieties under optimal conditions (control) or exposed to drought stress. (A) NR, (B) NiR, (C) GS, and (D) GOGAT. Error bars represents the mean ± SD. Different alphabets within the group represents significant differences among treatments according to Duncan multiple range test at P ˂ 0.05.

## Slide 8
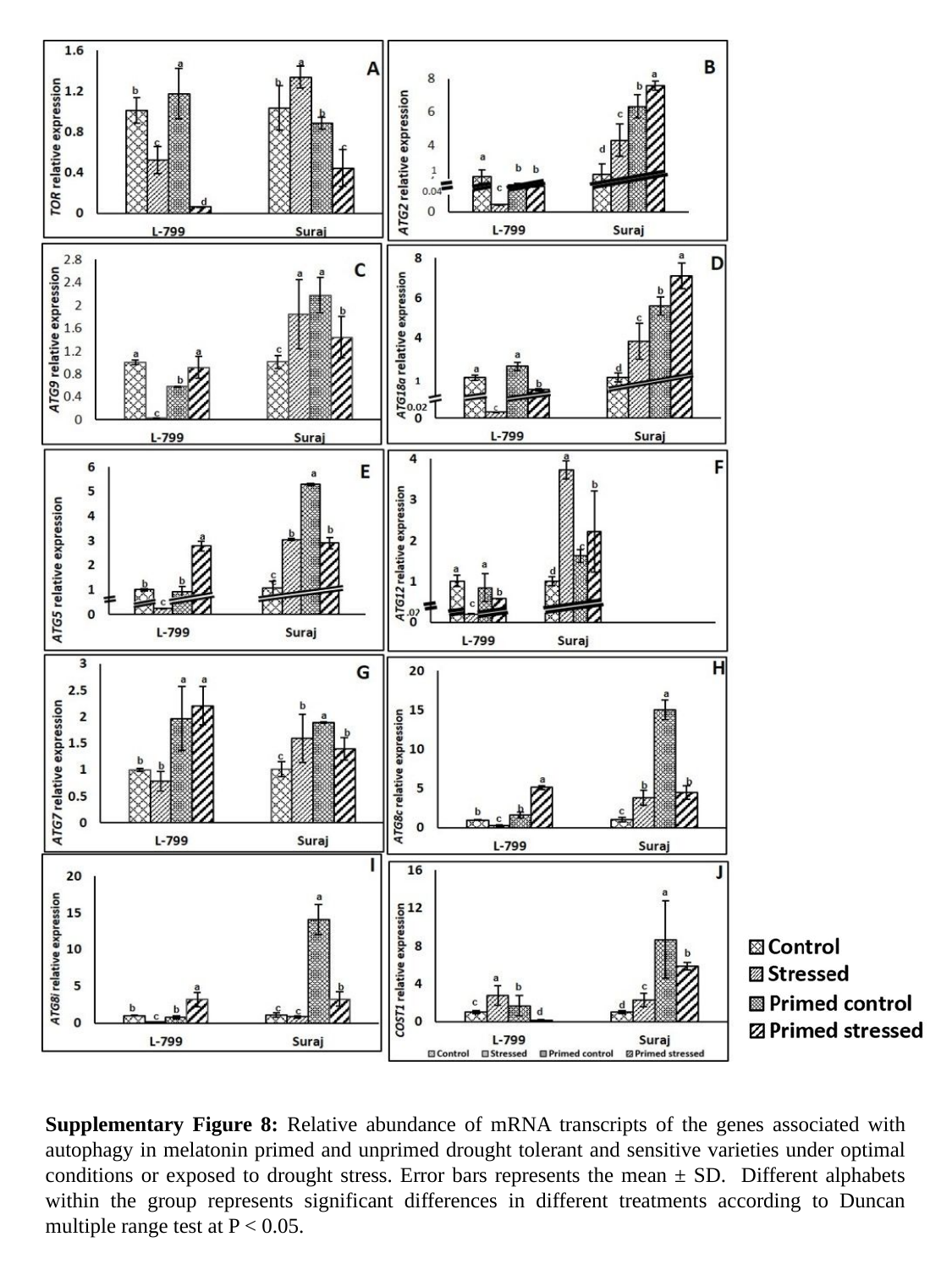

Supplementary Figure 8: Relative abundance of mRNA transcripts of the genes associated with autophagy in melatonin primed and unprimed drought tolerant and sensitive varieties under optimal conditions or exposed to drought stress. Error bars represents the mean ± SD. Different alphabets within the group represents significant differences in different treatments according to Duncan multiple range test at P ˂ 0.05.
