# Supplementary material for: Melatonin Mediated Differential Regulation of Drought Tolerance in Sensitive and Tolerant Varieties of Upland Cotton (Gossypium hirsutum L.)
Source: Front Plant Sci. 2022 Apr 4;13:821353. doi: 10.3389/fpls.2022.821353 (PMC9014207; doi:10.3389/fpls.2022.821353)
Supplement: Supplementary file 2 [file Table_1.DOCX]

| **Name of the genes** | **Forward primer (5’ to 3’)** | **Reverse primer (5’ to 3’)** |
| --- | --- | --- |
| *Actin4* | TGAGCAGGAACTGGAGACTG | GAGGACTTCTGGACAACGGA |
| *RBOH D* | GGCAGAAGGAACCAATAC | GGAGGACCACGTCATATT |
| *Cu-Zn SOD* | CCTTGCTGCCACTATTC | CAGTTTCCTGGGTCAAC |
| *Mn SOD* | CACCAGAAGAACCCTAAC | GCCATAGTCGTAAGGAAG |
| *CAT1* | AGTTCTACACCCGAGAG | GAGCATGGACCATATCAG |
| *cAPX* | CTTGAGGCAGGTGTTTAG | AGATGAGAGGGTTGGTAG |
| *gAPX* | CTGAGTTCCGCAAGTATG | GCTGAAGTGGGAGTAAAC |
| *GR* | CGAGTTACAGCGTCTTAC | GGATCCACAACCTTTCC |
| *PSII D1* | CTTCCTCTTGACCGAATCTG | GCTCCCTATTCAGTGCTATG |
| *Plastocyanin* | GTCTGGCTTTCATTCCCA | CGTCCTCATCGAACACAA |
| *Ferredoxin* | TAGAGGTGGAAGACGATGAG | CAGGGCAAGTCATGCAA |
| *rbs L* | CAGGCTGAAACAGGTGAA | CTCCCAATTCTCTAGCACAC |
| *FBP* | GCAGCAACAGCATCATCTCA | ACACGAGGGACTTGGAATCA |
| *SBP* | TGTAACGGGACGAGACCAAG | ATGGGTGCCAGGGATGT |
| *NR* | GACGAGACCGAGATGTAT | CCGATCATGCTTCTTAGC |
| *NiR* | GCCAAGAGATGTGATGAG | CGGTATCCAAGATCCCTA |
| *GS* | CCAGTGGGAGTATCAAGT | GGATCAAGGGAGAGAACA |
| *GOGAT* | GGTAGAAGTCTCCCAGAA | CATCCCATAGCTCCATTAG |
| *TOR* | GGCAATGAGGGACAATAC | GCATCCTTTCCAGTTCTC |
| *ATG2* | CCTACCGTCCAATCTTTG | CCTTCAGTACCCTCTGAT |
| *ATG9* | GGGATTCTCAGAGGTTATC | GCTTCAGGAGGTCTACTA |
| *ATG18a* | CTCCACATCTCCTTCAAC | GAAATCCCGGCGAAATA |
| *ATG5* | CCTCTTCAAATCCATCTCC | ATCAAAGGCGCTAACAG |
| *ATG12* | CTCACCAAACCCAGATG | CCCATGCCATAGAACAG |
| *ATG7* | CAGTATCCAGCCAAGAAG | CCATCGACTCTCTCTAGTA |
| *ATG8c* | GTATCCAGACAGAGTTCCT | CCCACAGTCAAATCAGATG |
| *ATG8i* | GTGGGTCAGTTCATTCAC | GAGACTTGCTGTTTGAGG |
| *COST1* | CCAGAAGAGAGTGAAACTG | CAAATCCGCAGGAAAGA |

**Supplementary Table 1:** List of primer sequences of the genes used for analysing their transcript levels using real-time PCR
